# Individual Differences in Developmental Trajectories of Global and Subcortical Brain Volumes Between Late Childhood and Late Adolescence: Findings From a 12‐Wave Neuroimaging Study

**DOI:** 10.1002/hbm.70348

**Published:** 2025-09-22

**Authors:** Chloe Carrick, William Frans Christiaan Baaré, Silia Vitoratou, Kathrine Skak Madsen, Delia Fuhrmann

**Affiliations:** ^1^ Department of Psychology Institute of Psychiatry, Psychology, and Neuroscience, King's College London London UK; ^2^ Danish Research Centre for Magnetic Resonance, Department of Radiology and Nuclear Medicine Copenhagen University Hospital ‐ Amager and Hvidovre Hvidovre Denmark; ^3^ Department of Biostatistics and Health Informatics Institute of Psychiatry, Psychology, and Neuroscience, King's College London London UK

**Keywords:** adolescence, brain development, cortex, individual differences, subcortical structures, white matter

## Abstract

Adolescence is characterised by protracted structural brain development, with different brain regions showing distinct developmental trajectories. While studies have identified average developmental trajectories, few have formally quantified individual differences in the developmental trajectories of global brain structures and subcortical regions across adolescence. Utilising the unique 12 waves of high temporal resolution MRI data from the Danish HUBU cohort (*N* = 90; ages seven to 21; 745 scans; on average 8.30 scans per participant) and nonlinear mixed modelling techniques, we examined both group and individual‐level patterns of volumetric change in global brain measures and subcortical regions. At the group level, cortical grey matter, total brain, caudate, putamen, accumbens, and thalamus volume decreased, while white matter, amygdala, hippocampus, and pallidum volume increased. We observed substantial interindividual variability in the rate of volumetric change in the caudate, as well as in the age at which cortical grey matter, white matter, and pallidum volumes changed most rapidly. For instance, the age of most rapid cortical volumetric decline varied by up to 7.5 years among individuals. Maturational trajectories also differed by sex. Our findings quantify overall trajectories, as well as individual and sex differences in volumetric development in subcortical and global brain volumes. Future research can build upon these findings to investigate the extrinsic and intrinsic factors that influence interindividual variations in developmental trajectories of adolescent brain structure, as well as how they relate to later‐life outcomes including mental health.

## Graphical abstract

Global and subcortical brain volumes demonstrate sex‐specific increases (white matter, hippocampus, amygdala, and pallidum) and decreases (cortical grey matter, total brain volume, caudate, putamen, accumbens, and thalamus) across adolescence. There are individual differences in the timing and pace of developmental trajectories.

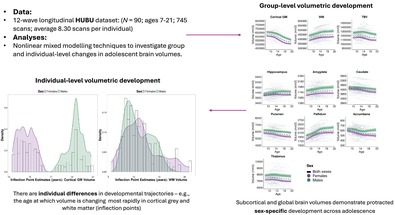

**Summary**

- Subcortical and global brain regions demonstrated dynamic development across adolescence, including volumetric increases (white matter, hippocampus, amygdala, and pallidum) and decreases (cortical grey matter, total brain volume, caudate, putamen, accumbens and thalamus).
- There were individual differences in the pace of maturation in the caudate and in the age of most rapid volumetric change in cortical grey matter, white matter, and the pallidum.
- Maturational trajectories of volume significantly differed by sex for all investigated brain regions.

## 1 Introduction

Adolescence, defined as the developmental phase between childhood and adulthood (10 and 24 years; Sawyer et al. 2018), is a period characterized by micro‐ and macrostructural brain changes (Bethlehem et al. 2022; Mills et al. 2021; Vijayakumar et al. 2018). These changes in brain structure are integral to outcomes in adulthood, including cognitive functioning and mental well‐being (Fuhrmann et al. 2015; Paus et al. 2008). Different cortical and subcortical regions show distinct maturational trajectories, with some brain regions maturing early and others showing protracted development. Average developmental changes across adolescence include S‐shaped nonlinear reductions in the thickness, surface area, and volume of cortical grey matter, nonlinear decreases in total brain volume, and nonlinear increases in white matter volume (Fuhrmann et al. 2022; Lebel et al. 2019; Mills et al. 2016; Tamnes et al. 2017; Vijayakumar et al. 2021).

The trajectories of subcortical grey matter development during adolescence are not fully understood. Longitudinal investigations of subcortical development report inconsistent findings for the direction and shape of volumetric change in each subcortical structure (Backhausen et al. 2024; Becht, Klapwijk, et al. 2020; Dennison et al. 2013; Goddings et al. 2014; Herting et al. 2018; Jones et al. 2023; Mills et al. 2014; Narvacan et al. 2017; Raznahan et al. 2014; Tamnes et al. 2013, 2018; van Drunen et al. 2024; Wierenga et al. 2014; Wierenga, Bos, et al. 2018; Zhou et al. 2021). The most common findings reported in these studies are volumetric decreases in the caudate, putamen, nucleus accumbens, and thalamus, occurring in parallel with increases in hippocampus and amygdala volume, although divergent findings have also been reported, including development in the opposite direction, as well as stability across the adolescent period (Backhausen et al. 2024; Dennison et al. 2013; Frere et al. 2020; Goddings et al. 2014; Herting et al. 2018; Jones et al. 2023; Raznahan et al. 2014; Tamnes et al. 2013; Wierenga et al. 2014; Wierenga, Bos, et al. 2018). Thus, our understanding of development in subcortical structures is more limited compared with cortical brain changes.

While prior research has focused on defining group‐level patterns of brain development, a growing body of evidence indicates substantial heterogeneity in individual developmental trajectories (Bottenhorn et al. 2023; Fuhrmann et al. 2022; Mills et al. 2021). By quantifying individual variations in structural brain development, we can begin to understand the factors influencing this heterogeneity, such as biological sex (Wierenga et al. 2022) and environmental factors like urbanicity and poverty (Ferschmann et al. 2022). Research shows that females mature earlier than males regarding cortical grey matter and white matter development (Frere et al. 2020; Lenroot et al. 2007; Peper et al. 2020) and that age trajectories of subcortical volume development can differ in direction and magnitude between the sexes (Backhausen et al. 2024; Dennison et al. 2013; Frere et al. 2020; Herting et al. 2018; Jones et al. 2023; van Drunen et al. 2024). Notably, adolescence is a period of heightened risk for the emergence of mental health disorders (Paus et al. 2008; Solmi et al. 2022), and research links individual differences in cortical grey matter (Bos, Peters, et al. 2018; Bos, Wierenga, et al. 2018; Kjelkenes et al. 2022; Noordermeer et al. 2017; Whittle et al. 2020), white matter (Kjelkenes et al. 2022; Muetzel et al. 2018), and subcortical limbic and striatal regions (Bos, Wierenga, et al. 2018; Mancini et al. 2020; Mattoni et al. 2021) to psychopathological symptoms. Individual differences in adolescent structural brain development are, therefore, increasingly viewed as a valuable source of insight into how deviations from normative development may be associated with the development of psychopathology (Becht and Mills 2020). However, most studies have been restricted in their ability to investigate individual differences in trajectories due to the lack of data points (< 3) and the limited resolution in temporal sampling in existing developmental neuroimaging datasets.

While it is possible to model individual change with two timepoints of data (King et al. 2018), for precise estimation of individual‐level trajectories, at least three data points are needed. Work using simulated data suggests that models with three or more timepoints can recover individual‐level growth parameters with increasing accuracy, and as such, it has been suggested that studies using two timepoints of data should focus on modeling group‐level patterns of change only (Parsons and McCormick 2024). While existing datasets generally average fewer than three waves of data per participant (Vijayakumar et al. 2018), large, prospective datasets with multiple waves are emerging (e.g., ABCD; Casey et al. 2018). Statistical methods that allow for modeling inter‐individual variability in nonlinear developmental trajectories of brain structure are necessary to capture trajectories accurately. Generalized Additive Mixed Models (GAMMs) can be used to quantify average developmental trajectories without predefining their expected shape, whilst accounting for individual‐level variance (Sørensen et al. 2021; Wood 2017). GAMMs have been increasingly used to model developmental changes in cortical grey matter (Tamnes et al. 2017) and subcortical structures (Backhausen et al. 2024; Herting et al. 2018; Jones et al. 2023; Wierenga, Bos, et al. 2018). Like GAMMs, nonlinear mixed models (NLMMs) can capture nonlinear maturational trajectories at the individual level but also estimate growth parameters, such as developmental asymptotes or growth rates (Davidian and Giltinan 2003). For instance, Fuhrmann et al. (2022) used a four‐parameter logistic function within the NLMM framework to characterize the S‐shaped maturational trajectory of cortical thickness from late childhood to late adolescence (ages 7 to 21 years) in the unique longitudinal HUBU cohort with up to 12 data points, showing considerable variability in the age at which cortical thinning was most rapid during adolescence, as indicated by the *inflection point* parameter (the Midpoint of Cortical Thinning).

The present study aims to characterize individual variability in the volumetric development of cortical grey matter, white matter, total brain volume, and the volumes of subcortical brain structures by leveraging nonlinear modeling techniques (GAMMs and NLMMs) and the single‐cohort HUBU dataset, which includes up to 12 repeated measurements of data spanning late childhood to late adolescence (Fuhrmann et al. 2022; Madsen et al. 2020). We hypothesized that global and subcortical brain volumes would show protracted development over the investigated developmental period, with developmental trajectories differing between brain structures and individuals. We further hypothesized that cortical grey matter volume would show an S‐shaped decrease, white matter volume a nonlinear increase, and total brain volume an initial nonlinear increase followed by a decrease across the investigated age range. Given the discrepancies in prior research regarding volumetric development in subcortical regions, we did not have any specific hypotheses about subcortical patterns of maturation; rather, we employed a data‐driven approach by using GAMMs to determine the shape of development in subcortical trajectories across adolescence. Furthermore, using NLMMs, we aimed to quantify the extent of individual differences in brain maturational trajectory parameters, such as the inflection point (i.e., age of most rapid volumetric change) and slope (i.e., rate of change), in each brain region across adolescence.

## 2 Materials and Methods

### 2.1 Participants and Study Design

**FIGURE 1:**
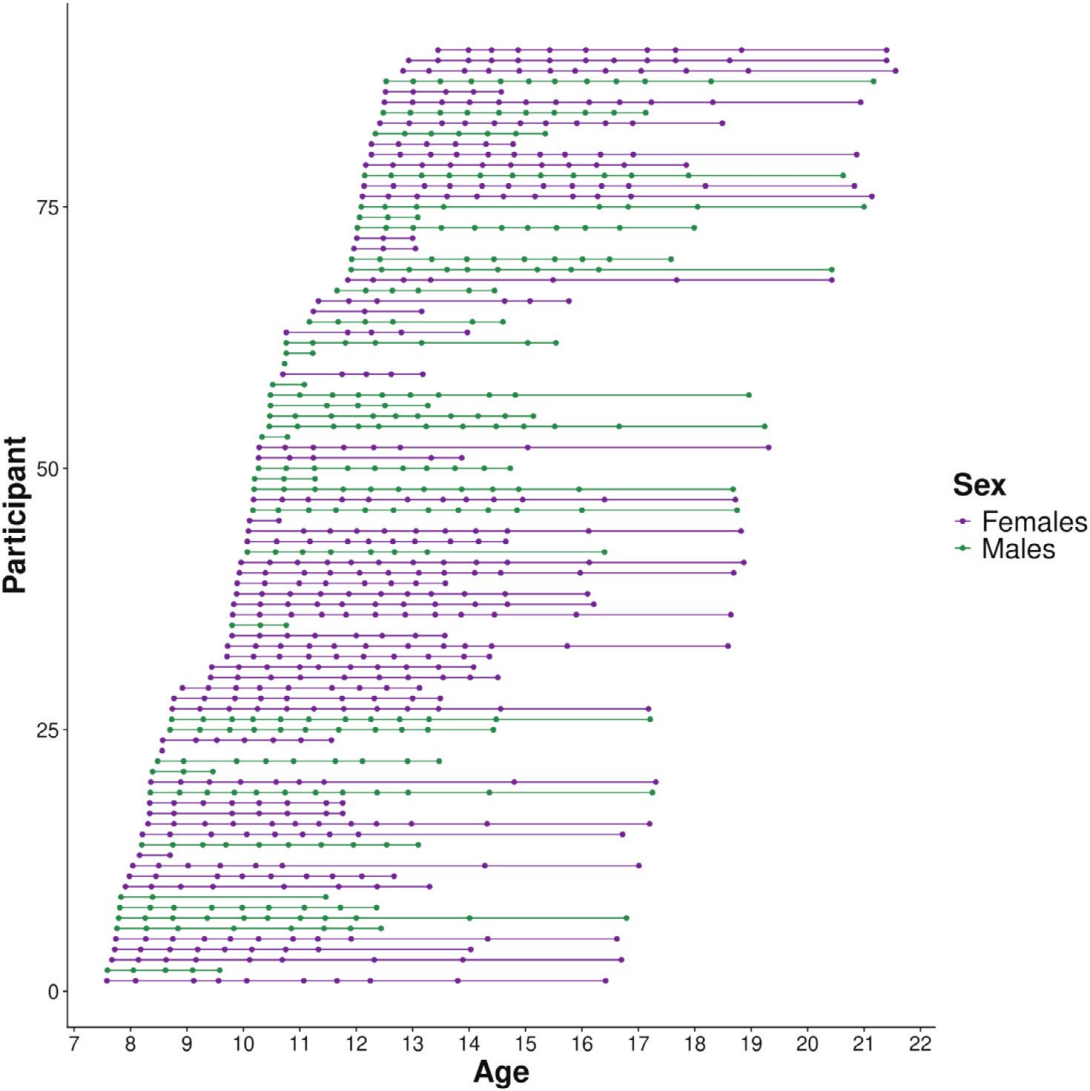
The timing of included scans for each participant (*N* = 90).

This study includes data from 90 typically developing children and adolescents (sex assigned at birth: 53 females, 37 males) enrolled in the longitudinal HUBU study (“Hjernens Udvikling hos Børn og Unge”: Brain Maturation in Children and Adolescents; Madsen et al. 2020). Participants were between 7.60 and 12.90 at baseline, and the entire study spanned the age range of 7.60 to 21.60 years. Participants were scanned up to 12 times, with six‐month intervals between the first 10 scans, one year between scans 10 and 11, and three years between scans 11 and 12 (Figure 1). Written consent was obtained from the parents of all participants before study initiation, and from the participants when they turned 18 years of age. The HUBU study was approved by the Ethical Committees of the Capital Region of Denmark (H‐KF‐01‐131/03 and H‐3‐2013‐037) and carried out in accordance with the Declaration of Helsinki.

Five participants (out of the original 95 enrolled in HUBU) were excluded from the current study because of incidental MRI findings (*N* = 1), receiving a psychiatric diagnosis after the initiation of the study (*N* = 2), or insufficient quality MRI scans (*N* = 2). For the 90 participants included in our final sample, we excluded an MRI session (i.e., scan) if it met the following criteria: the participant did not complete the T1 scan (two scans, two participants), the participant had metallic dental braces (31 scans,14 participants), the quality of the MRI scan was poor (31 scans, 22 participants), or the participant acquired a brain injury after baseline (nine scans, one participant). Participants were scanned on average 8.30 times (range = 1–12 scans, median = 9 scans). No exclusions were made based on the number of scans available per participant. In total, 745 valid MRI scans were used for statistical analyses in the current study. Data from the HUBU cohort has been used in prior longitudinal investigations of structural brain development (Fuhrmann et al. 2022; Madsen et al. 2020; Plachti et al. 2023).

### 2.2 Structural Imaging

#### 2.2.1 MRI Acquisition

Structural MRI scans were acquired using a 3T Siemens Magnetom Trio MR scanner (Siemens, Erlangen, Germany) and an eight‐channel head coil (Invivo, FL, USA). An MPRAGE sequence was used to acquire T1‐weighted images of the whole head (TR = 1550 ms, TE = 3.04 ms, matrix = 256 × 256, 192 sagittal slices, 1 × 1 × 1 mm^3^ voxels, acquisition time = 6:38). T2‐weighted images were obtained using a 3D turbo spin echo sequence (TR = 3000 ms, TE = 354 ms, FOV = 282 × 216, matrix = 256 × 196, 192 sagittal slices, 1.1 × 1.1 × 1.1 mm^3^ voxels, acquisition time = 8:29). Baseline images were inspected for incidental findings by a neuroradiologist. All raw images were visually assessed for quality and excluded if they were of inadequate quality.

#### 2.2.2 FreeSurfer Pre‐Processing and Extraction of Volume Estimates

T1‐ and T2‐weighted images were processed using the longitudinal pipeline of FreeSurfer version 6.0 (https://surfer.nmr.mgh.harvard.edu/; Dale et al. 1999; Dale and Sereno 1993; Reuter et al. 2012). Volumes, in mm^3^, were obtained for the hippocampus, amygdala, caudate, putamen, pallidum, accumbens area, and thalamus for the left and right hemispheres. Following the approach of prior investigations of subcortical volumetric development (Backhausen et al. 2024; Herting et al. 2018; Wierenga, Bos, et al. 2018), we averaged the volumes of the subcortical regions of interest (ROIs) across hemispheres. Further, *global* brain measures included cortical grey matter volume, white matter volume (referring to cerebral white matter i.e., excluding the cerebellar white matter), total brain volume, excluding the ventricles (labelled “BrainSegVolNotVent” in FreeSurfer output) and estimated Total Intracranial Volume (“eTIV” in FreeSurfer output). The latter was used in sensitivity analyses of sex differences in trajectories of volumetric development.

#### 2.2.3 Quality Control

Post‐processing quality control was carried out on FreeSurfer outputs according to the ENIGMA protocol (https://enigma.ini.usc.edu/protocols/imaging‐protocols/). We performed within‐ and between‐subjects statistical outlier detection for each ROI by fitting GAMMs adjusted for age, sex, and an age‐by‐sex interaction. Potential outliers were identified based on the extracted residual values from these models and flagged for further visual inspection. For between‐subjects outlier detection, we visually inspected ROI segmentations with residual values ≥ 2 SDs from the mean residual value across participants for that structure. For within‐subjects outlier detection, we inspected data points with residual values ≥ 2 SDs from the participant's mean residual value for each structure. Residuals were plotted as spaghetti plots across time, and data points with abrupt deviations from the individual trajectory were visually inspected. Data were excluded if the segmentation quality was deemed poor. Based on these checks, 20 scans (from two participants) were excluded for the accumbens, and analyses were conducted using 725 observations for this structure (88 participants). The Euler number, which reflects the topological complexity of the reconstructed cortical surface (Dale et al. 1999), was used as a proxy for participant movement and image quality (Rosen et al. 2018). The more holes in the initial cortical surface reconstruction, the more negative the Euler number, and the more likely the participant moved during image acquisition (Rosen et al. 2018). In our statistical analyses, the average Euler number across both hemispheres was used to model participant in‐scanner motion and image quality. This metric has also been used to index scan quality in previous studies with the HUBU cohort (Fuhrmann et al. 2022).

### 2.3 Statistical Analyses

All analyses were conducted in R version 4.4.2 (R Core Team 2024). Our analysis code is posted to the Open Science Framework and available here: https://osf.io/wujzs/.

#### 2.3.1 GAMMs

GAMMs were used to model the average developmental trajectories for each structure and to inform our choice of function to model trajectories via nonlinear mixed models (NLMMs). GAMMs can handle sparse longitudinal data with unequal numbers of time points and/or unequal intervals between time points. Furthermore, GAMMs capture the best relationship between a predictor and an outcome variable without needing to specify the expected shape of this relationship (Sørensen et al. 2021; Wood 2017). GAMMs were modelled using the *gamm* function from the mgcv package (version 1.9–1; Wood 2017). Visualisation of GAMMs was conducted using packages itsadug (version 2.4.1; van Rij et al. 2017) and ggplot2 (version 3.5.1; Wickham 2016).

Specifically, developmental change was modeled using a nonlinear smooth curve built using simpler basis functions (Sørensen et al. 2021). The number of basis functions determines the complexity of the smooth function and is specified using the k‐parameter. Here, we used a k‐parameter of 7, given that results from the k.check function and observed edf values indicated that this value was sufficiently large to capture the relationship between age and volumetric development for the investigated structures. We used thin plate regression splines as basis functions and restricted maximum likelihood to estimate the smoothing parameters, which penalize the complexity or ‘wiggliness’ of the curve, in line with the approach of prior literature (Madsen et al. 2020; Plachti et al. 2023; Sørensen et al. 2021). The estimation of smooth curves renders several test statistics (*F* value, *p*‐value), including an estimation of the effective degrees of freedom (edf), which indicates the degree of nonlinearity of the curve. A smooth curve with an edf of 1 indicates a linear relationship, edfs between 1 and 2 reflect a weakly nonlinear relationship, and an edf greater than 2 indicates nonlinearity in the smooth trajectory (Wood 2017; Zuur et al. 2009).

We included a random intercept for each participant in the GAMM analyses. Given that the aim of the GAMM analyses was to examine average developmental trajectories for each structure, we did not include additional random effects (e.g., random slopes or random smooths). This approach aligns with previous studies using GAMMs to model mean changes in brain volume across development (Backhausen et al. 2024; Wierenga, Bos, et al. 2018). Individual differences in trajectories were then estimated in the NLMMs. To examine whether including a random slope in analyses affected results, we reran our GAMM analyses, including a random slope for each participant (Table S1). Model estimates were similar across GAMMs with and without random slopes, with edf values differing less than 1 between analysis approaches (Table S1; Table 1).

Volume (y) was modelled as a smooth function of age $\left(s_{1}\left(\mathrm{age}\right)\right)$, a main effect of sex, holding age constant $\left(\beta_{1}\mathrm{sex}\right)$, a sex difference in the age trajectory (age × sex interaction: $s_{2}\left(\mathrm{age}\right)\left(\mathrm{sex}\right)$), a random intercept (u), and residual error (ε). $\beta_{0}$ represents the intercept:

$$y=\beta_{0}+\beta_{1}\mathrm{sex}+s_{1}\left(\mathrm{age}\right)+s_{2}\left(\mathrm{age}\right)\;\left(\mathrm{sex}\right)+u+\varepsilon$$

**FIGURE 2:**
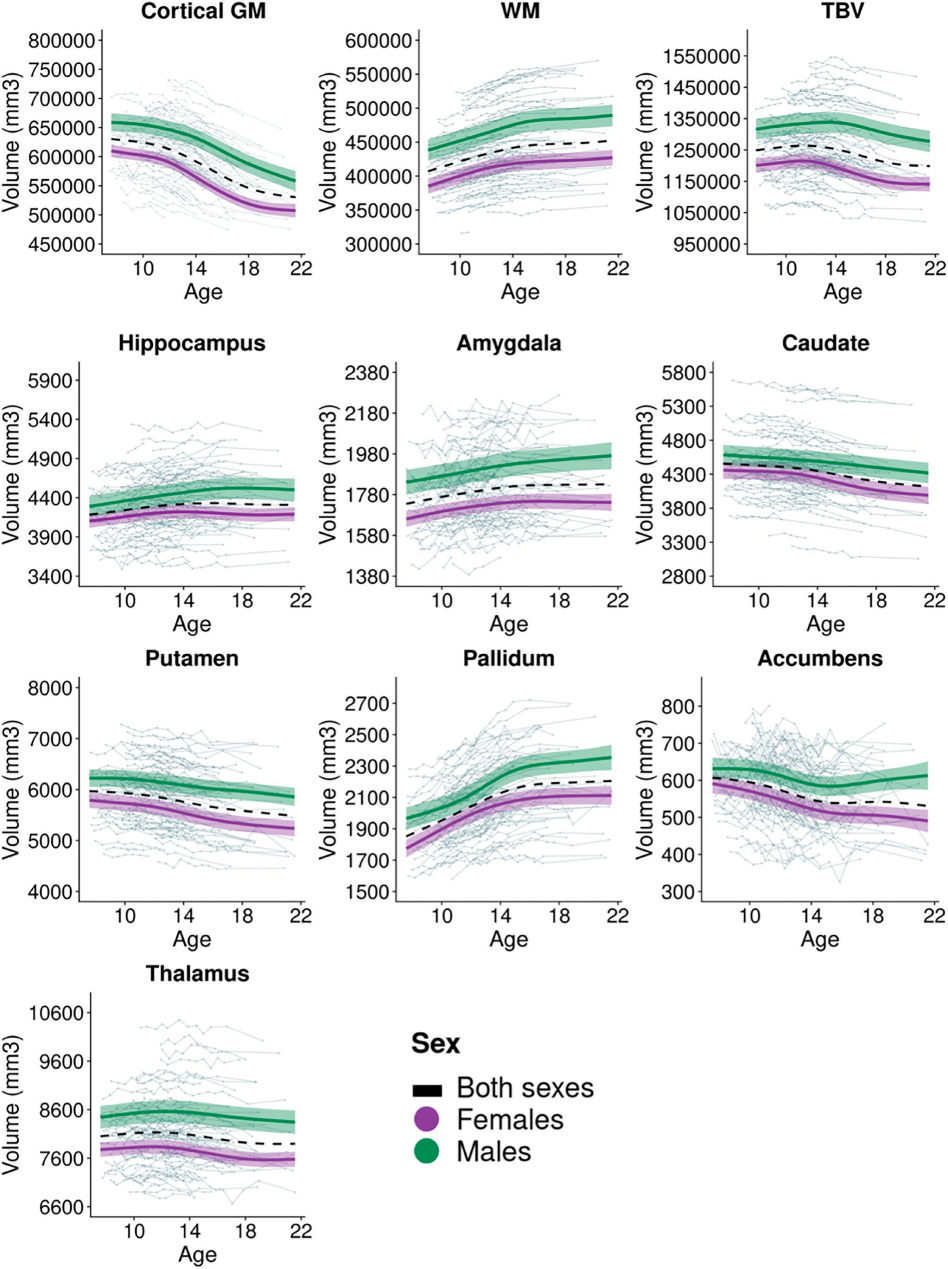
GAMM age trajectories for volumetric change in global brain measures and subcortical regions. Trajectories (with 95% confidence intervals) are plotted separately for females, males, and both sexes, and overlaid on individual trajectories for each participant. Cortical GM = cortical grey matter. WM = white matter. TBV = total brain volume.

To estimate interaction effects ($s_{2}\left(\mathrm{age}\right)\;\left(\mathrm{sex}\right)$), we coded sex as an ordered factor as recommended by prior studies (Backhausen et al. 2024; Herting et al. 2018; Sørensen et al. 2021). Additionally, and in line with prior literature (Backhausen et al. 2024; Herting et al. 2018), we conducted GAMMs in males and females separately to better understand potential sex differences in trajectories of volumetric development. These results are reported in the Supporting Information (Table S2) and visualised in Figure 2. Additionally, as a sensitivity analysis, we repeated our GAMMs to account for the five scanner software updates that occurred over the data collection for the HUBU project, including this variable as a main effect in analyses.

#### 2.3.2 NLMMs

Where the GAMMs indicated non‐linearity, we used NLMMs to further quantify trajectories of volumetric development. Trajectories were modelled using the saemix package (version 3.3; Comets et al. 2017). We modelled the relationship between age and volumetric development in each structure using three function transformations, each capturing a plausible trajectory: a logistic function capturing S‐shaped volume change, a logarithmic function capturing a decreasing rate of volumetric change over time, and a simple linear model characterising linear changes in volumetric development. We compared the model fit for each model using the Akaike Information Criterion (AIC; Akaike 1998) and the Bayesian Information Criterion (BIC; Schwarz 1978). Lower values indicate better model fit. We report group‐level and individual‐level parameter estimates for the best fitting of the three models for each brain volume measure. Following the approach of Fuhrmann et al. (2022), trajectories were first modelled using a four‐parameter logistic function:

$$y=A_{\text{lower}}+\frac{A_{\text{upper}}-A_{\text{lower}}}{1+{\left(\frac{x}{\text{Inflection}}\right)}^{-\text{Hill}}}+\varepsilon$$

This function characterises an S‐shaped trajectory in volumetric change and yields four parameter estimates: the lower asymptote ($A_{\text{lower}}$), reflecting minimal volume in mm^3^ , the upper asymptote ($A_{\text{upper}}$), reflecting maximal volume in mm^3^, the Hill, which indicates the slope of change in volume with increasing age, and the Inflection point. The inflection point corresponds to the x‐value (age) at which volume is changing most rapidly for an individual. We were interested in this parameter as an index of inter‐individual variability in brain maturation. The residual error is represented by ε. This four‐parameter logistic function is here‐in‐after referred to as the logistic function.

Volumetric change was next modeled using a logarithmic function:

y=a+b×logx+ε

This function models growth trajectories characterised by a decreasing growth rate over time (i.e., the rate of volumetric change decreases with increasing age). It yields two parameters: a corresponds to the intercept (this indicates volume when age = 1, and therefore was not interpreted, given it falls outside of the age range investigated in this study) and b reflects the rate of change in volume over time, with positive values indicating increasing volume with age. The b parameter was of interest to this work as an indicator of inter‐individual variability in volumetric development for each region.

Finally, a linear function was used to model maturation in each brain volume measure:

y=a+bx+ε

The parameter a represents the intercept, and b represents the slope of change. This slope parameter was used to index inter‐individual variability in brain volume maturation.

In each NLMM, volume was modeled as the dependent variable and age as the independent variable. Due to convergence issues when running models with global brain volume measures, values for cortical grey matter and white matter volume were rescaled by dividing by 100 before conducting the reported analyses. Variable rescaling has been proposed as a viable solution for addressing convergence problems with NLMMs, particularly when dealing with variables with large numerical values (Kiernan et al. 2012).

The precision with which each parameter was estimated in each model was assessed using the coefficient of variation (CV) defined as the ratio of the standard error over the mean. Values less than 20% indicate that the parameter was estimated with good precision (Reed et al. 2002). Model fit was further assessed by examining diagnostic plots of population and individual predictions for each model. A linear trend between predicted and observed values, with values aligned along the dotted red line, indicated a good model fit (see Figures S3 and S6). Furthermore, it should be noted that it is possible for the upper and lower asymptote estimates of the logistic model to correspond with *x*‐values (i.e., ages) that are outside of the included age range (before seven, or after 21 years of age). We have noted instances where this has occurred in Figures S7 and S8.

#### 2.3.3 NLMM Covariates

Sex (covariate of interest) and in‐scanner motion (covariate of no interest) were included as model covariates in this study. In the NLMM framework, covariates can be included for any of the main fixed effect parameters. This yields a maximum of eight covariates for the logistic model and four covariates for the linear and logarithmic model. Applying covariates to all model parameters increases the risk of overfitting, which can obscure the true statistical significance of observed effects (Bilger and Manning 2015). Thus, to avoid overfitting, we defined an optimal covariate structure for logistic, logarithmic, and linear models using forward‐stepwise model selection with cortical grey matter volume as the output variable (see Table S3 for results of the forward‐selection process).

For the logistic model, the upper and lower asymptotes, inflection point, and hill were allowed to differ between sexes. Additionally, in‐scanner motion, indexed by the Euler number, was included as a covariate for the hill parameter. For the logarithmic model, the growth parameter (b) was allowed to covary by sex and in‐scanner motion. Finally, for the linear model, sex was included as a covariate for the intercept and slope parameter. This covariate structure was applied to all reported covariate models. As a sensitivity analysis, models were re‐run without covarying for sex and/or in‐scanner motion. Additionally, we carried out sensitivity analyses covarying for scanner software version on the parameter of interest (i.e., inflection point or slope). Notably, the distribution of age was similar in males and females, suggesting that similar ages were sampled between sexes (Figure S2). A two‐sample Kolmogorov–Smirnov test further indicated that age distributions in males and females were not significantly different from one another (*D* = 0.04, *p* = 0.96). Furthermore, a linear mixed effects model indicated that in‐scanner motion did not differ by sex; *B* [SE] = −14.93 [10.55], *t* (86) = −1.42, *p* = 0.16.

## 3 Results

### 3.1 Global Brain Volume Development

#### 3.1.1 Nonlinear Decreases in Cortical Grey Matter Volume

Cortical grey matter volume decreased nonlinearly between 7 and 21 years, as indicated by the GAMM (edf > 2; Table 1; Table S2). Visually, this trajectory was S‐shaped at the group level (Figure 2). AIC and BIC values in the NLMM also indicated that the S‐shaped logistic model captured the volumetric development of cortical grey matter better than the linear or logarithmic model (see Table S4 for fit indices for each model). Cortical grey matter volume was high in childhood, with an upper asymptote of approximately 610,541 mm^3^, reaching a lower asymptote of approximately 508,972 mm^3^ later in development (Table 2; Figure 2). The inflection point was estimated at 14.52 years, indicating the average age at which cortical grey matter volume was decreasing most rapidly during this developmental period. Predicted cortical grey matter volume as a logistic function of age is presented in Figure S1. The CV values for the upper and lower asymptote, inflection point, and hill were all below 20%, indicating that these parameters were estimated with precision. Diagnostic plots indicate that this model fit the data well (Figure S3A).

**TABLE 1:** GAMM estimates for age and sex effects on global and subcortical brain volumes.

Metric/structure	Effect	Statistics			
Cortical grey matter	Main effect	Estimate	SE	*t*	*p*
	Sex	42,427.50	6200.46	6.84	< 0.001
	**Trajectory**	**edf**	**Ref df**	***F***	***p***
	S(age)	5.55	5.55	656.39	< 0.001
	S(age): sex	4.56	4.56	21.37	< 0.001

White matter	Main effect	Estimate	SE	*t*	*p*
	Sex	38,738.46	6734.25	5.75	< 0.001
	**Trajectory**	**edf**	**Ref df**	***F***	***p***
	S(age)	5.30	5.30	489.25	< 0.001
	S(age): sex	4.79	4.79	33.82	< 0.001

Total brain volume	Main effect	Estimate	SE	*t*	*p*
	Sex	92,883.47	13,595.11	6.83	< 0.001
	**Trajectory**	**edf**	**Ref df**	***F***	***p***
	S(age)	5.54	5.54	212.27	< 0.001
	S(age): sex	4.89	4.89	39.43	< 0.001

Hippocampus	Main effect	Estimate	SE	*t*	*p*
	Sex	166.25	53.59	3.10	0.002
	**Trajectory**	**edf**	**Ref df**	***F***	***p***
	S(age)	4.48	4.48	23.41	< 0.001
	S(age): sex	4.02	4.02	18.96	< 0.001

Amygdala	Main effect	Estimate	SE	*t*	*p*
	Sex	128.68	23.58	5.46	< 0.001
	**Trajectory**	**edf**	**Ref df**	***F***	***p***
	S(age)	3.72	3.72	35.99	< 0.001
	S(age): sex	1.15	1.15	8.43	0.004

Caudate	Main effect	Estimate	SE	*t*	*p*
	Sex	166.10	69.37	2.39	0.02
	**Trajectory**	**edf**	**Ref df**	***F***	***p***
	S(age)	5.19	5.19	269.40	< 0.001
	S(age): sex	4.07	4.07	27.66	< 0.001

Putamen	Main effect	Estimate	SE	*t*	*p*
	Sex	371.05	81.27	4.57	< 0.001
	**Trajectory**	**edf**	**Ref df**	***F***	***p***
	S(age)	4.74	4.74	246.37	< 0.001
	S(age): sex	1.00	1.00	57.58	< 0.001

Pallidum	Main effect	Estimate	SE	*t*	*p*
	Sex	112.09	30.64	3.66	< 0.001
	**Trajectory**	**edf**	**Ref df**	***F***	***p***
	S(age)	4.84	4.84	308.31	< 0.001
	S(age): sex	4.81	4.81	21.29	< 0.001

Accumbens	Main effect	Estimate	SE	*t*	*p*
	Sex	46.56	12.61	3.69	< 0.001
	**Trajectory**	**edf**	**Ref df**	***F***	***p***
	S(age)	4.73	4.73	55.57	< 0.001
	S(age): sex	1.71	1.71	11.97	< 0.001

Thalamus	Main effect	Estimate	SE	*t*	*p*
	Sex	527.86	92.58	5.70	< 0.001
	**Trajectory**	**edf**	**Ref df**	***F***	***p***
	S(age)	5.02	5.02	62.01	< 0.001
	S(age): sex	1.82	1.82	12.84	< 0.001

*Note:* Sex represents the difference in the intercept for males compared with females. S(age) represents the smooth function of age for females. S(age): sex represents an age‐by‐sex interaction – the difference in the age trajectory of males compared with females. For each main effect, estimate, standard error (SE), *t*‐value, and *p*‐value are reported. Effective degrees of freedom (edf), reference degrees of freedom (Ref df), *F*‐statistic, and *p*‐value are reported for each smooth function.

**TABLE 2:** NLMM parameter estimates for cortical grey matter and white matter volume development.

	Estimate	SE	CV (%)	*p*
**Cortical grey matter**				
Lower Asymptote	5089.72	57.63	1.13	—
b Sex (Lower Asymptote)	−232.79	279.63	120.12	0.41
Upper Asymptote	6105.41	63.18	1.03	—
b Sex (Upper Asymptote)	488.24	97.48	19.97	< 0.001
Inflection	14.52	0.24	1.68	—
b Sex (Inflection)	4.92	1.09	22.19	< 0.001
Hill	−5.94	0.72	12.12	—
b Sex (Hill)	2.10	0.70	33.21	0.003
b Motion (Hill)	0.01	0.004	37.62	0.008
**White matter**				
Lower Asymptote	3736.34	56.32	1.50	—
b Sex (Lower Asymptote)	289.28	102.59	35.50	0.005
Upper Asymptote	4555.92	76.24	1.70	—
b Sex (Upper Asymptote)	544.69	117.46	21.60	< 0.001
Inflection	13.91	0.95	6.80	—
b Sex (Inflection)	−1.64	1.40	85.10	0.24
Hill	3.89	0.54	13.90	—
b Sex (Hill)	0.17	0.55	322.50	0.76
b Motion (Hill)	0.003	0.002	61.60	0.10

*Note:* Estimates are presented from the logistic model. Females are the reference group for sex covariate parameters. SE = Standard Error. CV = Coefficient of Variation.

#### 3.1.2 Nonlinear Increases in White Matter Volume

The GAMM showed that white matter volume changed non‐linearly between ages 7 and 21 (edf > 2; Table 1; Table S2), increasing rapidly until mid‐adolescence, with this increase slowing down after that (Figure 2).

The logistic model was the best‐fitting NLMM of white matter volumetric development (Table S4). Starting in childhood at a lower asymptote of approximately 373,634 mm^3^, volume increased to an upper asymptote of approximately 455,592 mm^3^ later in development (Table 2). White matter volume was increasing most rapidly at 13.91 years, as indexed by the average inflection point. Group‐level parameters were estimated with precision (CVs < 20%; Table 2), and diagnostic plots indicate that this model fit the data well (Figure S3B). Predicted white matter volume as a logistic function of age is presented in Figure S1.

#### 3.1.3 Nonlinear Changes in Total Brain Volume

Total brain volume decreased nonlinearly between ages 7 and 21, as indicated by the GAMM (edf > 2; Table 1; Table S2). The greatest decreases in volume occurred from mid‐adolescence onwards, leveling off at the end of the included age range (Figure 2).

The logistic model, as compared to the logarithmic and linear models, was the best‐fitting NLMM for total brain volume development (Table S4). Nevertheless, diagnostic models indicated that the logistic model fitted total brain volume development poorly (see clustering in Figure S4). Furthermore, the hill parameter had a very high CV value (100.74; Table S5), suggesting it was estimated imprecisely. Consequently, we did not interpret the logistic model further, and subsequent discussions concerning total brain volume development are solely based on GAMM results.

#### 3.1.4 Individual Differences in Global Brain Volume Development

**FIGURE 3:**
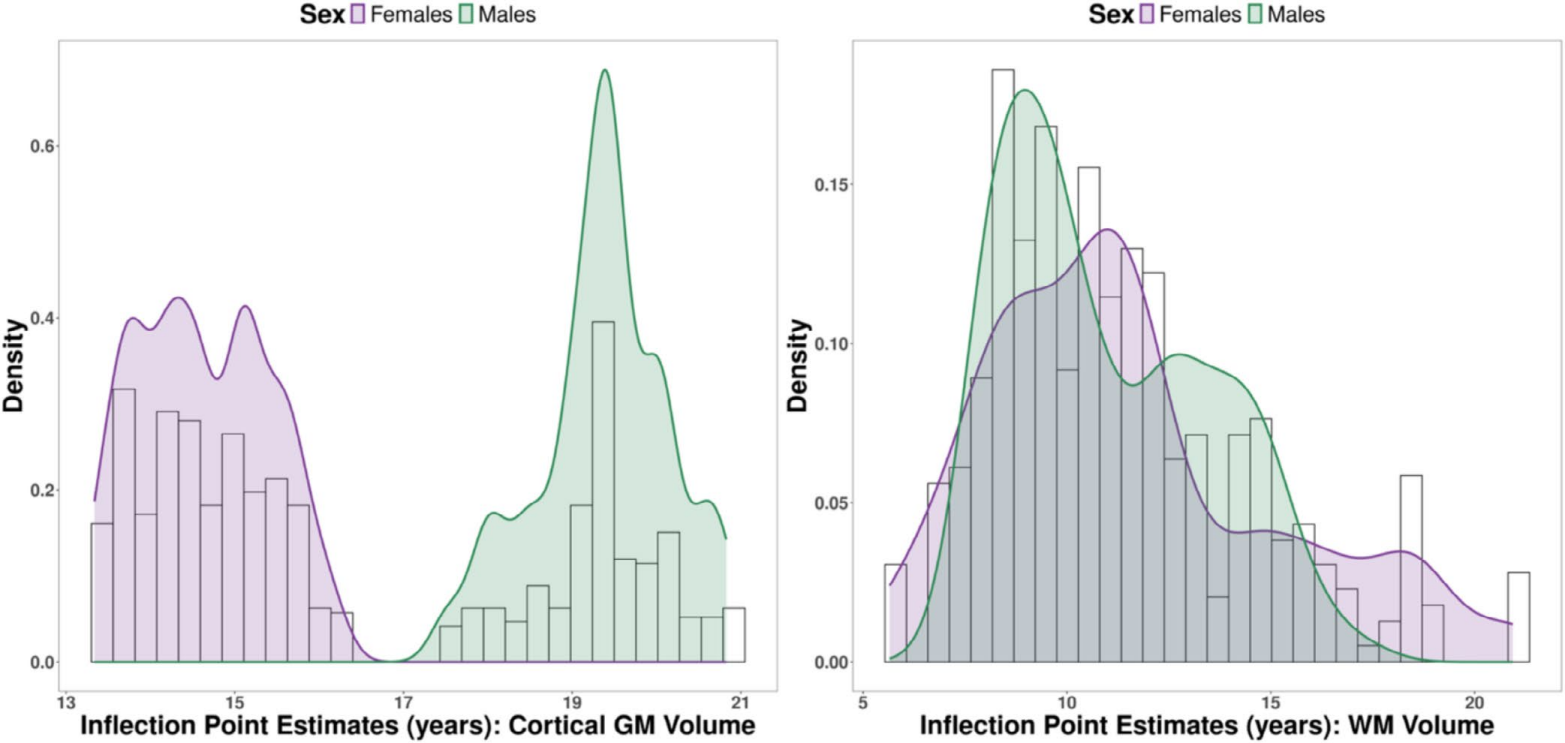
Density plots of individual inflection point estimates for cortical grey matter (GM) and white matter (WM) volume indicate substantial inter‐individual variability in age of the most rapid volumetric changes during adolescence.

To investigate individual differences in developmental trajectories, we examined the variation in the inflection point estimates extracted from the logistic NLMM. Figure 3 displays a density plot of individual inflection point estimates from the sample. There were substantial inter‐individual differences in the age of the most rapid cortical grey matter volume change during adolescence, with estimated individual inflection points for cortical grey matter ranging from 13.34 to 20.83 years. The inflection points for white matter volume also varied considerably between individuals (Figure 3), ranging from 7.64 to 20.94 years. Five individuals had inflection point estimates that were slightly younger than the minimum age included in this sample (range = 5.65–7.40 years). To check the reliability of these estimates, we examined individual diagnostic plots for these participants. These plots indicated that for most individuals, observations aligned with the predicted trajectories (Figure S5).

#### 3.1.5 Sex Differences in Global Brain Volume Development

There were significant sex differences in the developmental trajectories of cortical grey matter (significant sex and age‐by‐sex interaction effects in GAMMs; Table 1). Females, as compared to males, had lower cortical grey matter volumes and showed steeper volume decreases during mid‐adolescence that levelled off in late adolescence, while males showed a more gradual decrease across adolescence (Figure 2). The group‐level estimates from the logistic NLMM aligned with these findings; females had lower cortical grey matter than males in late childhood and late adolescence, a faster rate of change, as indexed by the hill, and an earlier inflection point (by 4.92 years on average, SE = 1.09 years; b Sex(Inflection) in Table 2). However, it should be noted that the CV values for estimates of sex differences in the hill and inflection point were above 20%, indicating noise in the estimation of these trajectories. Figure 3 shows a distinct bimodal distribution of inflection point estimates, with female inflection points clustering earlier than males (average female inflection point = 14.56 years; average male inflection point = 19.38 years).

GAMM estimated age trajectories of white matter development also showed significant sex and age‐by‐sex interaction effects (Table 1). Females had lower white matter volumes than males across adolescence, and volume increases in females appeared to level off earlier than in males (Figure 2). This was in line with logistic model estimates, indicating that females had lower white matter volume in childhood and adulthood (Table 2). Notably, the CVs for estimates of sex differences in white matter volume trajectories were above 20%, indicating noise in the estimation of these parameters, likely due to lower sample sizes in sub‐groups.

For total brain volume, GAMMs also indicated significant sex and age‐by‐sex interaction effects (Table 1). Females, as compared to males, had lower total brain volumes across adolescence and showed steeper decreases in mid‐adolescence (Figure 2).

### 3.2 Subcortical Volume Development

#### 3.2.1 Increases in Hippocampus and Amygdala Volume

Hippocampus and amygdala volumes increased between ages 7 and 21 (Figure 2). While GAMM estimates indicated that these volumetric increases were nonlinear (edfs > 2; Table 1; Table S2), AIC and BIC values in the NLMMs indicated that the linear function captured the volumetric change in these structures better than the logistic or logarithmic function (Table S4). In line with GAMMs, NLMMs also indicated increases in volume across adolescence (Table 3).

**TABLE 3:** NLMM parameter estimates for subcortical structures fit with a linear function.

Structure	Estimate	SE	CV (%)	*p*
**Hippocampus**				
Intercept	4116.00	50.87	1.24	—
b Sex (Intercept)	58.60	80.19	136.88	0.47
Slope	6.50	2.01	31.05	—
b Sex (Slope)	13.40	3.32	24.79	< 0.001
**Amygdala**				
Intercept	1639.66	22.48	1.37	—
b Sex (Intercept)	115.55	35.68	30.87	0.0012
Slope	6.44	1.07	16.59	—
b Sex (Slope)	5.32	1.77	33.23	0.0026
**Caudate**				
Intercept	4673.60	66.7	1.43	—
b Sex (Intercept)	45.90	104.48	227.45	0.66
Slope	−31.00	1.83	5.89	—
b Sex (Slope)	14.40	3.00	20.78	< 0.001
**Putamen**				
Intercept	6185.50	79.37	1.28	—
b Sex (Intercept)	317.50	124.54	39.22	0.01
Slope	−45.70	2.19	4.78	—
b Sex (Slope)	16.30	3.61	22.26	< 0.001
**Thalamus**				
Intercept	8095.50	89.47	1.11	—
b Sex (Intercept)	530.70	140.76	26.52	< 0.001
Slope	−24.40	3.12	12.78	—
b Sex (Slope)	17.10	5.15	30.11	< 0.001

*Note:* Females are the reference group for sex covariate parameters. SE = Standard Error. CV = Coefficient of Variation.

Intercept and slope parameters for the amygdala were estimated with good precision (CVs > 20%; Table 3); however, the slope of volumetric change in the hippocampus was estimated with poor precision (CV > 20%, Table 3). Diagnostic plots for all NLMMs for subcortical structures are presented in Figure S6. Clustering in the diagnostic plots of predicted vs. observed values for group‐level volumetric development in the hippocampus and amygdala suggests poor model fits.

#### 3.2.2 Decreases in Caudate, Putamen, and Thalamus Volume

The caudate, putamen, and thalamus decreased in volume from late childhood to late adolescence (Figure 2). GAMM estimates indicated that these decreases were nonlinear (edfs > 2; Table 1; Table S2). However, AIC and BIC values in the NLMMs indicated that a linear function captured age‐related volumetric development better than the logistic or logarithmic model (Table S4). The NLMMs replicated the overall developmental trend, indicating decreasing volume in these structures over adolescence (Table 3). CV values indicated that the intercept and slope parameters from the linear model were estimated with good precision for each structure (Table 3). However, the diagnostic plot of predicted vs. observed values for group‐level volumetric development in the thalamus and putamen indicated clustering, suggesting imprecise model fits (Figure S6).

#### 3.2.3 Non‐Linear Change in Pallidum and Accumbens Volume

The pallidum and accumbens demonstrated nonlinear volumetric change across adolescence in the GAMMs (edfs > 2; Table 1; Table S2). The pallidum increased, while the accumbens decreased until mid‐adolescence, after which the change gradually leveled off (Figure 2). In agreement with these findings, the logistic model was the best‐fitting model of volumetric development for both structures (Table S4).

On average, pallidum volume increased in an S‐shaped pattern from childhood to later in development, starting at 1741.55 mm^3^ and reaching an upper asymptote of 2154.30 mm^3^. The average inflection point was estimated at 10.99 years. The upper and lower asymptotes, inflection point, and hill were estimated with good precision (CVs < 20%; Table 4).

**TABLE 4:** NLMM parameter estimates for subcortical structures best fit with a logistic function.

Structure	Estimate	SE	CV (%)	*p*
**Pallidum**				
Lower Asymptote	1741.55	37.01	2.13	—
b Sex (Lower Asymptote)	192.86	52.08	27.01	< 0.001
Upper Asymptote	2154.30	35.60	1.65	—
b Sex (Upper Asymptote)	230.59	58.31	25.29	< 0.001
Inflection	10.99	0.35	3.23	—
b Sex (Inflection)	1.61	0.49	30.35	< 0.001
Hill	5.17	0.82	15.93	—
b Sex (Hill)	1.74	0.81	46.60	0.03
b Motion (Hill)	0.0016	0.0038	241.70	0.68
**Accumbens**				
Lower Asymptote	435.33	27.06	6.22	—
b Sex (Lower Asymptote)	133.73	32.46	24.27	< 0.001
Upper Asymptote	674.27	31.15	4.62	—
b Sex (Upper Asymptote)	44.17	54.12	122.51	0.41
Inflection	11.44	1.28	11.20	—
b Sex (Inflection)	−2.90	2.06	71.15	0.16
Hill	−0.43	0.63	146.85	—
b Sex (Hill)	−0.81	1.08	133.33	0.45
b Motion (Hill)	0.01	0.005	39.58	0.012

*Note:* Females are the reference group for sex covariate parameters. SE = Standard Error. CV = Coefficient of Variation.

Accumbens volume was high in childhood, starting with an upper asymptote of 674.27 mm^3^ and decreasing to a lower asymptote of 435.33 mm^3^ later in development. The inflection point was estimated at 11.44 years (Table 4). While the upper and lower asymptotes and inflection point had CVs < 20%, the hill parameter had a CV value of 146.85%, indicating that this parameter was estimated with poor precision. Given that this parameter had the highest estimated CV value of any group‐level parameter in the investigated structures, we conducted a sensitivity analysis of accumbens development in a model without covariates and examined the precision with which the hill parameter was estimated (Table S6). In this model, the CV value for the hill (*Estimate* = −2.20) reduced to 22.70%. However, given that this value is still above 20%, estimations of this parameter should be interpreted with caution in models with and without covariates. Logistic model predictions of accumbens volume are presented in Figure S8.

#### 3.2.4 Individual Differences in Subcortical Development

**FIGURE 4:**
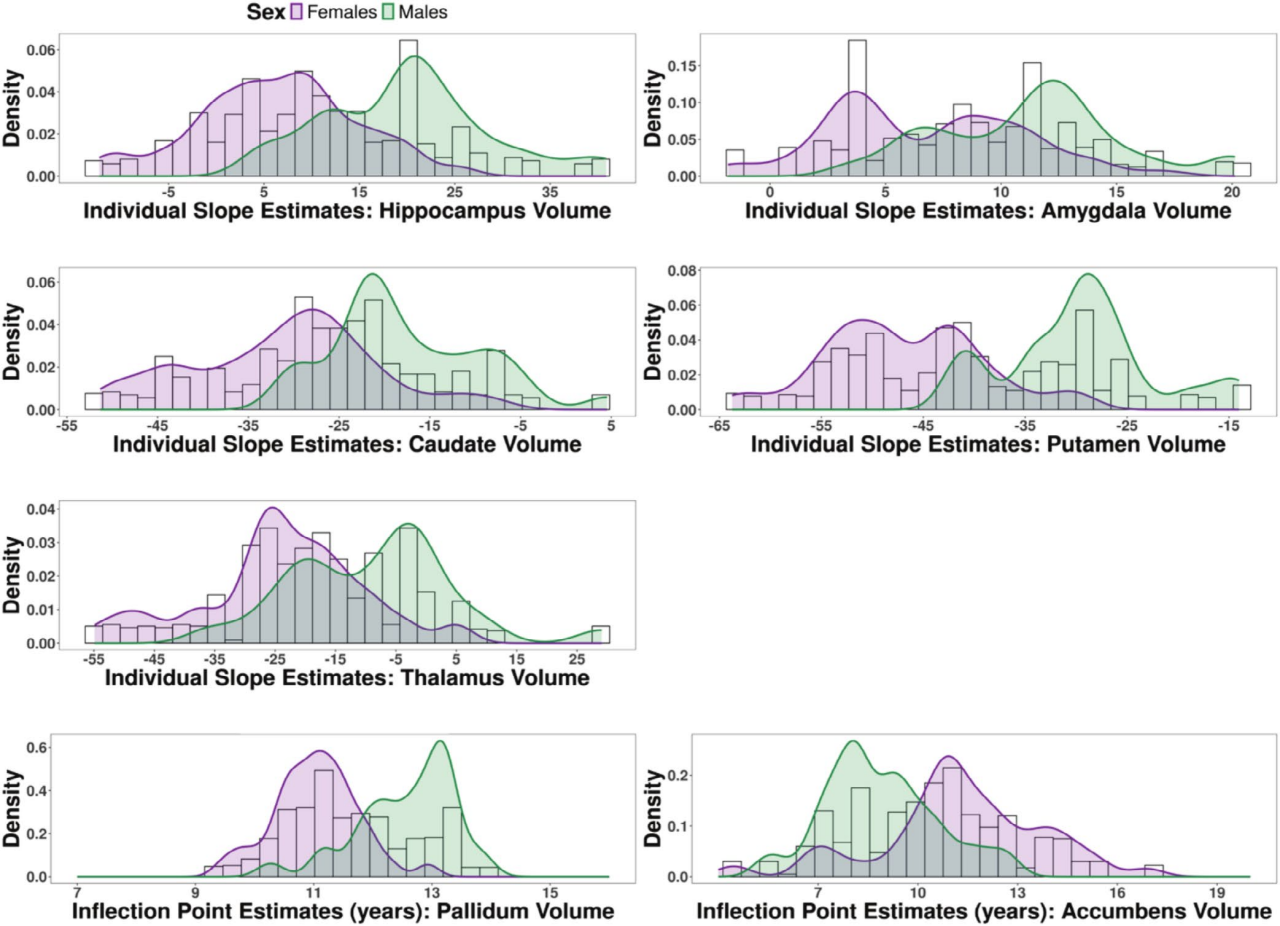
Density plots of individual slope estimates for hippocampus, amygdala, caudate, putamen, and thalamus volumes, and inflection point estimates for pallidum and accumbens volume. The density plots indicate substantial inter‐individual variability in development during adolescence, though there were potential issues with model fit for hippocampus, amygdala, putamen, thalamus, and accumbens development. The plots further indicate potential sex differences in slopes and inflection points, though the sex differences were estimated with poor precision (i.e., high CVs).

To investigate individual differences in developmental trajectories, we examined the variation in NLMM parameter estimates. For each subcortical structure where the linear function was the best fit (hippocampus, amygdala, caudate, putamen, thalamus), we extracted individual slope estimates, observing substantial heterogeneity between individuals (e.g., caudate slope estimate range = −51.33 − 4.54; see Figure 4 for a density plot of the slope estimates). However, given that diagnostic plots suggested poor model fit for the hippocampus, amygdala, putamen, and thalamus (Figure S6), individual differences for slope estimates for these structures should be interpreted with caution.

For the pallidum and accumbens, we extracted each participant's inflection point estimate for volumetric development. Pallidum inflection points ranged from 9.45 to 13.86 years, indicating individual differences in the age at which volumetric growth in the pallidum is most rapid (Figure 4). For the accumbens, among the age range sampled, the inflection points ranged from 7.82 to 16.89 years (Figure 4). Sixteen participants had accumbens inflection point estimates younger than the minimum age in this sample (range = 4.46–7.51). Of these, five had three observations of data or less, with the age ranges of these observations clustering at earlier ages, which may have given rise to the earlier inflection point estimates for these individuals (Figure S9). Individual prediction plots indicated that most observations aligned with predicted trajectories for the remaining 11 individuals. However, given the high CV value for the hill of accumbens development (Table 4), individual differences in developmental trajectories for this structure should be interpreted cautiously.

#### 3.2.5 Sex Differences in Subcortical Development

GAMM modelling revealed significant sex and age‐by‐sex interaction effects for all subcortical ROIs, indicating that males had generally larger volumes than females and that females and males differed in their estimated developmental trajectories.

NLMM parameter estimates suggested that males, as compared to females, had faster rates (i.e., steeper slopes) of hippocampal and amygdala volume increase (Table 3; b Sex (Slope)). For the caudate, putamen, and thalamus, males displayed slower rates of volume decrease compared with females, as indicated by the flatter slope estimates (Table 3).

NLMM model parameter estimates for the pallidum suggested that males, as compared to females, had larger volumes across the investigated age period, a later inflection point, and a steeper hill estimate (Table 4). The average male inflection point was 12.63 years, while the average female inflection point was 11.02 years. The only significant sex difference in accumbens trajectories was observed for the lower asymptote, with males exhibiting larger volumes in adulthood (Table 4).

Notably, the CV values of estimated sex differences in trajectory parameters for all subcortical ROIs were above 20%, indicating poor precision, likely due to the lower sample sizes in subgroups. Furthermore, it should be noted that the age by sex interaction in the GAMM of amygdala development was no longer significant when including a random slope in analyses (*p* = 0.05; Table S1). Thus, these apparent sex differences should be interpreted cautiously.

### 3.3 Sensitivity Analyses

#### 3.3.1 No Covariate Models

The above statistical analyses were repeated without covarying for sex or in‐scanner motion. These models identified that the best fits were the same as in the main analysis for all structures except for the amygdala, for which the logistic instead of the linear model gave the best fit (Table S7). Parameter estimates for the latter model are reported in Table S6.

#### 3.3.2 Models Controlling for eTIV

To better understand sexual dimorphisms in developmental trajectories of global and subcortical brain volumes, we repeated our GAMM analyses controlling for eTIV in all models, as females generally have smaller intracranial volumes than males. We hereby follow recommendations in prior literature (Vijayakumar et al. 2018). After controlling for eTIV, sex differences in developmental trajectories remained significant for all investigated brain volumes (significant age‐by‐sex interaction effect; Table S8). This indicates that observed patterns of volumetric maturation across subcortical and global brain structures differed between males and females, irrespective of any differences in intracranial volume. However, the main effect of sex was no longer significant for white matter, hippocampus, caudate, putamen, pallidum, and accumbens volume (Table S8), indicating that sex differences in these brain structures were not specific and driven by differences in intracranial volume. The latter was not the case for cortical grey matter, total brain, amygdala, and thalamus volumes, which, on average, were larger in males than females, even after controlling for eTIV.

#### 3.3.3 Models Controlling for Scanner Software Updates

To assess whether scanner software updates that occurred over the course of the HUBU data collection influenced our results, we reran our main models, including scanner software version as an additional covariate for the inflection point and slope of development for structures estimated with precision using NLMMs (cortex, white matter, pallidum, and caudate). Additionally, we included scanner software version as a main effect in our GAMM analyses for each structure. Software‐related variance was not associated with inflection points in the cortex, white matter, or pallidum (*p* values > 0.05). These estimates remained similar to the inflection points estimated in our main NLMMs, differing by less than a year (cortex = 13.85 years; white matter = 14.86 years; pallidum = 11.22 years). While software version was significantly associated with the slope of caudate development (*p* = 0.004), the direction of change remained the same, with the slope estimate reducing in magnitude from −31.00 to −22.60. The main effect of scanner software version was nonsignificant for all structures except for the hippocampus and pallidum (Table S9). However, the edf values remained virtually unchanged for these structures (Hippocampus edf = 4.43, Pallidum edf = 4.84) and the age by sex interaction effect remained significant. Thus, taken together, these results suggest that software‐related variance did not account for our findings.

### 3.4 Relationship Between Baseline Levels and Magnitude of Change in Brain Volumes

For structures that were modelled with precision using NLMMs (cortex, white matter, pallidum, and caudate), we conducted exploratory correlational analyses to examine whether baseline volumes, as reflected in the upper or lower asymptotes of development, were associated with the rate of change in those structures over time. Absolute values were used for the hill and slope of development to reflect the magnitude of change in volume across adolescence.

We found a significant negative association between the upper asymptote of the cortex and the hill of development (*r*(88) = −0.34, *p* = 0.001), indicating that those with greater cortical volume in late childhood demonstrated less volumetric change across adolescence. Furthermore, we found a significant positive association between the lower asymptote of the pallidum and the hill of development (*r*(88) = 0.40, *p* < 0.001), indicating that those with greater baseline levels of pallidum volume also had greater increases in volume across adolescence. There were no significant associations between baseline levels and rates of change in white matter or caudate volume. These findings indicate that, for the cortex and pallidum, the initial size of these regions is related to subsequent patterns of volumetric development across adolescence. These associations could reflect, for instance, the presence of subpopulations with divergent developmental trajectories. Alternatively, they could represent a modeling artefact (Xu et al. 2012). Additional research is needed to prospectively examine the relationship between developmental milestones across the adolescent period in larger and diverse samples.

## 4 Discussion

This study characterised inter‐individual variability in developmental trajectories of global and subcortical brain volumes, using a unique longitudinal neuroimaging dataset with up to 12 waves spanning ages 7 to 21 years. It also addressed the knowledge gap regarding the shape of maturational trajectories of subcortical brain volumes between late childhood and late adolescence. We elucidated substantial inter‐individual variability in the maturational trajectories of global and regional brain measures. Inflection point estimates for cortical grey matter, white matter, and pallidum development differed by several years between individuals, highlighting heterogeneity in the age at which these structures underwent the most rapid volumetric change across adolescence. Additionally, we observed individual differences in the rate of volumetric change in the caudate. There were also sex differences in the maturational trajectories; for example, females showed earlier cortical grey matter maturation than males, with an average inflection point occurring ~5 years earlier than in males. At the group level, all investigated brain structures demonstrated protracted development in adolescence, with varied patterns of volumetric increases (white matter, hippocampus, amygdala, and pallidum) and decreases (cortical grey matter, total brain volume, caudate, putamen, accumbens and thalamus). Our work highlights that adolescence is a period of dynamic brain maturation, with individuals displaying marked heterogeneity in developmental trajectories of brain structures. It also sheds light on distinct patterns of volumetric change in subcortical regions.

As hypothesized, we observed considerable inter‐individual variation in estimated maturational trajectories. The age at which cortical grey matter, white matter, and the pallidum showed the most rapid volume changes (inflection point) varied by several years between individuals; for example, it differed by up to 7.5 years in cortical grey matter. Additionally, we found substantial inter‐individual differences in the rate of volumetric change in the caudate, with developmental changes differing in both magnitude and direction between individuals (caudate slope values ranged from −51.33 to 4.54). Although we also observed individual differences in developmental trajectories of hippocampus, amygdala, putamen, thalamus, and accumbens volumes, potential model fit issues precluded further interpretation of these findings. Together, our findings support a growing body of evidence indicating that patterns of structural development are heterogeneous between individuals (Becht, Wierenga, et al. 2020; Bottenhorn et al. 2023; Fuhrmann et al. 2022; Mills et al. 2021). Furthermore, given that previous research has primarily focused on individual differences in the rate of change (slope) in brain structure, the present investigation extends these findings by elucidating significant variation in the phase of maturation, highlighting differences in the age at which individuals reach the specific developmental milestone of the most rapid volumetric change (Olson et al. 2025). The observed variation in maturational patterns of brain structures invites investigations of how individual intrinsic (e.g., genetic makeup or puberty) and/or extrinsic factors (e.g., environmental contexts, including home, school, and neighbourhood factors) may impact these individual differences in development, as well as investigating to which extent this variability is associated with, for example, mental health outcomes later in life (Farah 2017; Ferschmann et al. 2022; Foulkes and Blakemore 2018). Future cohorts with larger sample sizes, spanning wide age ranges, are necessary to investigate these questions with sufficient power.

Notably, inflection point estimates for white matter volume development demonstrated considerable variability (ranging between 7.64 and 20.94 years)—this was more pronounced than for other regions. Five individuals had white matter inflection point estimates that were earlier than the included age range. White matter volume increases over a large age range, throughout childhood, adolescence, and into early adulthood (Bethlehem et al. 2022; Lebel and Deoni 2018). It is thus possible that individuals demonstrate greater variability in the age at which white matter volumetric increases were most rapid compared with other structures. Future studies, including an age range with earlier ages, including the age of peak growth rate of white matter volume across the lifespan—2.4 years—as identified by Bethlehem et al. (2022), will yield more clarity regarding inflection points in white matter volume.

Our findings substantiate and extend current knowledge of group‐level patterns of development in global brain structures. As hypothesised and in line with prior literature (Fuhrmann et al. 2022; Mills et al. 2016; Tamnes et al. 2017), cortical grey matter, white matter, and total brain volume developed nonlinearly, with decreasing cortical grey matter and total brain volume and increasing white matter volume across the investigated age range. Specifically, our findings advance our understanding of cortical grey matter and white matter volume development by revealing that cortical grey matter volume underwent the most rapid change at 14.52 years of age and white matter at 13.91 years of age. As cortical grey matter volume is primarily determined by cortical thickness and surface area (Panizzon et al. 2009), and we previously observed accelerated cortical thinning at approximately 14 years in the same cohort (Fuhrmann et al. 2022), the observed age of peak cortical grey matter volume change is likely driven by changes in cortical thinning.

We observed volumetric increases in the hippocampus, amygdala, and pallidum, which occurred alongside decreases in the volume of the caudate, putamen, accumbens, and thalamus across adolescence. The shape of average GAMM trajectories for the different subcortical structures observed in our study is similar to those found in a multi‐sample study of three cohorts with three waves spanning the age range 8 to 22 years (Herting et al. 2018). The overall *direction* of change was consistent across GAMM and NLMM analyses. However, some uncertainty remains regarding the *shape* of the observed developmental trajectories. While GAMM models indicate nonlinear patterns of change in all subcortical structures, NLMM analyses revealed that the linear model captured the volumetric development of the hippocampus, amygdala, putamen, caudate, and thalamus better than the nonlinear logistic and logarithmic functions. Longitudinal studies employing GAMMs to examine developmental trajectories mainly report nonlinear developmental trends (Backhausen et al. 2024; Herting et al. 2018; Jones et al. 2023; Wierenga, Bos, et al. 2018; Zhou et al. 2021). Longitudinal studies using linear mixed‐effects models have reported linear (Dennison et al. 2013; Frere et al. 2020; van Drunen et al. 2024) as well as nonlinear (Goddings et al. 2014; Narvacan et al. 2017; Raznahan et al. 2014; Wierenga et al. 2014) changes. The discrepancies between GAMMs and NLMMs in the current study may result from the different estimators used. It is possible that the logistic, logarithmic, and linear functions in the NLMM analyses were not able to capture the “true” developmental trajectories in the hippocampus, amygdala, caudate, putamen, and thalamus. Large prospective datasets, such as the ABCD study, are emerging and will provide an opportunity for future investigations to replicate the shapes of trajectories reported here across the adolescent period when more time points are available and a larger age range is assessed.

We found significant sex differences in developmental trajectories consistent with prior literature on global brain structures (Frere et al. 2020; Lenroot et al. 2007; Mills et al. 2016; Peper et al. 2020; Wierenga, Sexton, et al. 2018) and subcortical regions (Backhausen et al. 2024; Dennison et al. 2013; Frere et al. 2020; Herting et al. 2018; Raznahan et al. 2014; van Drunen et al. 2024). On average, males showed larger global brain and subcortical volumes than females. However, when accounting for differences in intracranial volumes, males only had, on average, larger relative cortical grey matter, total brain, amygdala, and thalamus volumes. Similar to GAMMs, NLMM analyses consistently indicated that the maturational trajectories of volumetric development significantly differed between the sexes. However, the precision of estimated sex differences in model parameters was limited, as indicated by CVs larger than 20%. This is likely due to the relatively small sample sizes of the female and male subgroups. Nevertheless, NLMM analyses suggest that females underwent earlier rapid changes in cortical grey matter and pallidum volume, consistent with prior findings of earlier and greater cortical and subcortical volume changes in females (Mills et al. 2021). This earlier maturation may mirror earlier female pubertal timing (Goddings et al. 2019; Vijayakumar et al. 2021). Future research is warranted that uses larger developmental samples to examine sex differences in structural brain maturation, including sexual dimorphisms in developmental trajectories, as well as sex differences in developmental variability (Wierenga, Sexton, et al. 2018).

This study has several strengths and limitations. The use of flexible nonlinear mixed modelling approaches and a dataset with high temporal resolution sampling, averaging eight scans per individual, allowed for precise estimation of individual differences in developmental trajectories between ages 7 and 21 years. This study is one of the few to formally quantify inter‐individual heterogeneity in both global and subcortical brain developmental trajectories from late childhood to late adolescence. However, our findings should be viewed in light of several limitations. Firstly, the sociodemographic homogeneity of the HUBU cohort limits the generalizability of the present findings. Future research should replicate the present results in larger and more diverse sociodemographic samples, such as the ABCD study. The small sample size in sex subgroups is an additional limitation of the present investigation. However, it is worth noting that statistical power in NLMMs is not only dependent on sample size but also the number and spacing of repeated measurements (Roy and Ette 2005), which is particularly dense in the present sample. Nevertheless, due to low sample sizes in sex subgroups, observed sexual dimorphisms in developmental trajectories should be interpreted with caution. Future work that replicates these findings in larger samples is warranted to assess the robustness of the results reported here. Furthermore, discrepancies between GAMMs and NLMMs in the current study, as well as imprecisions in model fit for the hippocampus, amygdala, putamen, thalamus, accumbens, and total brain volume, suggest that the logistic, logarithmic, and linear functions used in the NLMM analyses were not always able to capture the “true” developmental trajectories of change in these structures. Future work with larger samples is needed to determine the optimal functional forms to index developmental change in these structures across adolescence. Moreover, there were several individuals for whom estimated inflection point values for white matter and accumbens development were outside the investigated age range, and investigating a broader age range may have provided more precise estimates of the inflection points for these brain regions. Finally, it is worth noting that the accumbens is a difficult region to segment, with previous research indicating potential issues with the reliability of accumbens volume estimates (Mills et al. 2021). This may have given rise to noise in the estimation of these trajectories.

## 5 Conclusion

The present study provides novel insight into inter‐individual variability in structural brain development during adolescence by identifying individual differences in the timing and pace of maturation of global and subcortical brain volumes. We provide evidence of substantial inter‐individual variability in the age at which individuals reach the developmental milestone of the most rapid volumetric change during adolescence within different brain structures (e.g., differing by up to 7.5 years between individuals for cortical grey matter). Furthermore, this study provides and extends knowledge on group‐level patterns of developmental trajectories for subcortical regions. Future research is needed to study how individual intrinsic (e.g., biological sex, genetic makeup or pubertal processes) and extrinsic factors (e.g., environmental contexts, including home, school, and neighbourhood factors) shape individual variations in global and regional brain maturation, and how these variations in trajectories are related to mental health outcomes. Additionally, it supports the development, testing, and optimisation of longitudinal brain data analysis methods.

## Supporting information


**Data S1:** Supplementary Information.

## Data Availability

Individual‐level data for the HUBU Study is confidential. The code to replicate analyses has been provided on Open Science Framework: https://osf.io/wujzs/.
