# Supplementary material for: Individual Differences in Developmental Trajectories of Global and Subcortical Brain Volumes Between Late Childhood and Late Adolescence: Findings From a 12‐Wave Neuroimaging Study
Source: Hum Brain Mapp. 2025 Sep 22;46(14):e70348. doi: 10.1002/hbm.70348 (PMC12451839; doi:10.1002/hbm.70348)
Supplement: Supplementary file 1 — Data S1: Supplementary Information. [file HBM-46-e70348-s001.docx]

**Supplementary Materials**

**Supplementary Table 1A.**

GAMM estimates for age and sex effects on global and subcortical brain volumes, including a random slope per participant.

| **Metric/structure** | **Effect** | | **Statistics** | | | |
| --- | --- | --- | --- | --- | --- | --- |
| **Cortical grey matter** | **Main effect** | | **Estimate** | **SE** | ***t*** | ***p*** |
|  | Sex | 42238.60 | | 6225.71 | 6.78 | <.001 |
|  | **Trajectory** | **edf** | | **Ref df** | ***F*** | ***p*** |
|  | S(age) | 5.60 | | 5.60 | 207.47 | <.001 |
|  | S(age): sex | 4.85 | | 4.85 | 16.24 | <.001 |
| **White matter** | **Main effect** | **Estimate** | | **SE** | ***t*** | ***p*** |
|  | Sex | 38320.88 | | 6730.25 | 5.69 | <.001 |
|  | **Trajectory** | **edf** | | **Ref df** | ***F*** | ***p*** |
|  | S(age) | 5.48 | | 5.48 | 148.64 | <.001 |
|  | S(age): sex | 5.03 | | 5.03 | 16.65 | <.001 |
| **Total brain volume** | **Main effect** | **Estimate** | | **SE** | ***t*** | ***p*** |
|  | Sex | 92376.40 | | 13633.16 | 6.78 | <.001 |
|  | **Trajectory** | **edf** | | **Ref df** | ***F*** | ***p*** |
|  | S(age) | 5.60 | | 5.60 | 87.90 | <.001 |
|  | S(age): sex | 5.09 | | 5.09 | 24.81 | <.001 |
| **Hippocampus** | **Main effect** | **Estimate** | | **SE** | ***t*** | ***p*** |
|  | Sex | 164.15 | | 53.83 | 3.05 | .002 |
|  | **Trajectory** | **edf** | | **Ref df** | ***F*** | ***p*** |
|  | S(age) | 4.56 | | 4.56 | 13.18 | <.001 |
|  | S(age): sex | 4.54 | | 4.54 | 8.60 | <.001 |
| **Amygdala** | **Main effect** | **Estimate** | | **SE** | ***t*** | ***p*** |
|  | Sex | 128.34 | | 23.48 | 5.47 | <.001 |
|  | **Trajectory** | **edf** | | **Ref df** | ***F*** | ***p*** |
|  | S(age) | 3.74 | | 3.74 | 19.16 | <.001 |
|  | S(age): sex | 1.00 | | 1.00 | 3.77 | 0.05 |
| **Caudate** | **Main effect** | **Estimate** | | **SE** | ***t*** | ***p*** |
|  | Sex | 164.61 | | 69.47 | 2.37 | .02 |
|  | **Trajectory** | **edf** | | **Ref df** | ***F*** | ***p*** |
|  | S(age) | 5.33 | | 5.33 | 72.77 | <.001 |
|  | S(age): sex | 4.65 | | 4.65 | 12.38 | <.001 |
| **Putamen** | **Main effect** | **Estimate** | | **SE** | ***t*** | ***p*** |
|  | Sex | 370.56 | | 80.94 | 4.58 | <.001 |
|  | **Trajectory** | **edf** | | **Ref df** | ***F*** | ***p*** |
|  | S(age) | 4.76 | | 4.76 | 94.75 | <.001 |
|  | S(age): sex | 1.00 | | 1.00 | 21.88 | <.001 |
|  |  |  | |  |  |  |
| **Pallidum** | **Main effect** | **Estimate** | | **SE** | ***t*** | ***p*** |
|  | Sex | 107.84 | | 31.31 | 3.44 | <.001 |
|  | **Trajectory** | **edf** | | **Ref df** | ***F*** | ***p*** |
|  | S(age) | 5.03 | | 5.03 | 83.19 | <.001 |
|  | S(age): sex | 4.85 | | 4.85 | 11.96 | <.001 |
| **Accumbens** | **Main effect** | **Estimate** | | **SE** | ***t*** | ***p*** |
|  | Sex | 46.55 | | 12.49 | 3.73 | <.001 |
|  | **Trajectory** | **edf** | | **Ref df** | ***F*** | ***p*** |
|  | S(age) | 4.81 | | 4.81 | 32.47 | <.001 |
|  | S(age): sex | 1.00 | | 1.00 | 8.58 | .003 |
| **Thalamus** | **Main effect** | **Estimate** | | **SE** | ***t*** | ***p*** |
|  | Sex | 526.51 | | 92.71 | 5.68 | <.001 |
|  | **Trajectory** | **edf** | | **Ref df** | ***F*** | ***p*** |
|  | S(age) | 5.11 | | 5.11 | 33.73 | <.001 |
|  | S(age): sex | 1.00 | | 1.00 | 7.32 | .01 |

**Supplementary Table 1B.**

GAMM estimates for age trajectories in males and females separately, including a random slope per participant.

|  | **Females** | | | | **Males** | | | |
| --- | --- | --- | --- | --- | --- | --- | --- | --- |
| **Structure** | **edf** | **Ref df** | ***F*** | ***p*** | **edf** | **Ref df** | ***F*** | ***p*** |
| Cortical grey matter | 5.57 | 5.57 | 201.90 | <.001 | 4.88 | 4.88 | 98.08 | <.001 |
| White matter | 5.43 | 5.43 | 163.41 | <.001 | 5.45 | 5.45 | 135.28 | <.001 |
| Total brain volume | 5.55 | 5.55 | 83.96 | <.001 | 5.11 | 5.11 | 40.10 | <.001 |
| Hippocampus | 4.60 | 4.60 | 17.12 | <.001 | 4.45 | 4.45 | 16.62 | <.001 |
| Amygdala | 3.70 | 3.70 | 18.92 | <.001 | 2.63 | 2.63 | 20.34 | <.001 |
| Caudate | 5.25 | 5.25 | 77.98 | <.001 | 3.26 | 3.26 | 18.48 | <.001 |
| Putamen | 4.72 | 4.72 | 96.43 | <.001 | 3.86 | 3.86 | 32.54 | <.001 |
| Pallidum | 4.97 | 4.97 | 93.51 | <.001 | 5.23 | 5.23 | 77.01 | <.001 |
| Thalamus | 4.89 | 4.89 | 25.66 | <.001 | 4.25 | 4.25 | 11.77 | <.001 |
| Accumbens | 4.05 | 4.05 | 37.85 | <.001 | 4.25 | 4.25 | 10.03 | <.001 |

**Supplementary Table 2.**

GAMM estimates for age trajectories in males and females separately.

|  | **Females** | | | | **Males** | | | |
| --- | --- | --- | --- | --- | --- | --- | --- | --- |
| **Structure** | **edf** | **Ref df** | ***F*** | ***p*** | **edf** | **Ref df** | ***F*** | ***p*** |
| Cortical grey matter | 5.50 | 5.50 | 648.24 | <.001 | 4.75 | 4.75 | 300.02 | <.001 |
| White matter | 5.22 | 5.22 | 505.24 | <.001 | 5.16 | 5.16 | 454.21 | <.001 |
| Total brain volume | 5.48 | 5.48 | 201.12 | <.001 | 4.91 | 4.91 | 58.90 | <.001 |
| Hippocampus | 4.47 | 4.47 | 26.34 | <.001 | 3.71 | 3.71 | 49.25 | <.001 |
| Amygdala | 3.60 | 3.60 | 38.11 | <.001 | 2.64 | 2.64 | 42.10 | <.001 |
| Caudate | 5.09 | 5.09 | 264.63 | <.001 | 2.70 | 2.70 | 93.79 | <.001 |
| Putamen | 4.67 | 4.67 | 232.81 | <.001 | 4.04 | 4.04 | 69.89 | <.001 |
| Pallidum | 4.77 | 4.77 | 317.70 | <.001 | 5.02 | 5.02 | 248.63 | <.001 |
| Thalamus | 4.90 | 4.90 | 57.58 | <.001 | 4.07 | 4.07 | 20.26 | <.001 |
| Accumbens | 3.98 | 3.98 | 57.40 | <.001 | 4.34 | 4.34 | 15.55 | <.001 |

*Note*: Effective degrees of freedom (edf), reference degrees of freedom (Ref df), *F*-statistic, and *p*-value are reported for each smooth function.

**Supplementary Table 3.**

Forward stepwise selection to determine the optimal covariate model in the NLMMs with cortical grey matter volume as the output variable. Tables A, B, and C present results for the logistic, logarithmic, and linear models respectively.

**A. Logistic model**

| **Covariate** | **1^st^ iteration** | **2^nd^ iteration** | **3^rd^ iteration** | **4^th^ iteration** | **5^th^ iteration** |
| --- | --- | --- | --- | --- | --- |
| sex: lower asymptote | 0 | - | - | - | - |
| sex: upper asymp | 0.000000660 | 0.0000036 | - | - | - |
| sex: MCT | 0 | - | - | - | - |
| sex: Hill | 0.00082 | 0.0024 | 0.000054 | - | - |
| motion: lower asymp | 0.78 | 0.74 | 0.29 | 0.24 | 0.11 |
| motion: upper asymp | 0.32 | 0.2 | 0.39 | 0.39 | 0.18 |
| motion: MCT | 0.11 | 0.67 | 0.66 | 0.94 | 0.58 |
| motion: Hill | 0.0015 | 0.0019 | 0.0026 | 0.0079 | - |

*Note:* This process begins with no covariates in the model. The *p*-value of each covariate is then checked when added to the model one at a time. In each iteration, the covariate parameter with the lowest *p*-value less than 0.05 is kept in the model (red cell).

**B. Logarithmic model**

| **Covariate** | **1^st^ iteration** | **2^nd^ iteration** | **3^rd^ iteration** |
| --- | --- | --- | --- |
| sex: intercept | 0.49 | 0.85 | 0.8 |
| motion: intercept | 0.70 | 0.6 | 0.85 |
| sex: growth | 0.00000000000046 | - | - |
| motion: growth | 0.09 | 0.002 | - |

**C. Linear model**

| **Covariate** | **1^st^ iteration** | **2^nd^ iteration** | **3^rd^ iteration** |
| --- | --- | --- | --- |
| sex:intercept | 0.000019 | 0.0062 | - |
| motion:intercept | 0.47 | 0.56 | 0.54 |
| sex: slope | 0.00000034 | - | - |
| motion: slope | 0.08 | 0.096 | 0.095 |

**Supplementary Table 4.**

NLMM model fit for different structures.

| **Structure** | **Model** | **AIC** | **BIC** |
| --- | --- | --- | --- |
| **Cortical grey matter** | ***Logistic*** | ***8936.36*** | ***8971.36*** |
|  | Logarithmic | 9513.23 | 9530.73 |
|  | Linear | 9237.30 | 9254.80 |
| **White matter** | ***Logistic*** | ***7892.997*** | ***7927.995*** |
|  | Logarithmic | 8039.03 | 8056.53 |
|  | Linear | 8082.98 | 8100.48 |
| **Total brain volume** | ***Logistic*** | ***6371.17*** | ***6406.17*** |
|  | Logarithmic | 6592.34 | 6609.84 |
|  | Linear | 6435.62 | 6453.12 |
| **Hippocampus** | Logistic | 8508.995 | 8543.99 |
|  | Logarithmic | 8587.98 | 8605.48 |
|  | ***Linear*** | ***8500.26*** | ***8517.76*** |
| **Amygdala** | Logistic | 7694.34 | 7729.34 |
|  | Logarithmic | 7757.05 | 7774.55 |
|  | ***Linear*** | ***7693.39*** | ***7710.89*** |
| **Putamen** | Logistic | 8924.12 | 8959.12 |
|  | Logarithmic | 8979.12 | 8996.62 |
|  | ***Linear*** | ***8901.89*** | ***8919.39*** |
| **Caudate** | Logistic | 8485.38 | 8520.38 |
|  | Logarithmic | 8447.61 | 8465.11 |
|  | ***Linear*** | ***8272.43*** | ***8289.93*** |
| **Thalamus** | Logistic | 9331.89 | 9366.89 |
|  | Logarithmic | 9320.73 | 9338.22 |
|  | ***Linear*** | ***9232.49*** | ***9249.99*** |
| **Pallidum** | ***Logistic*** | ***7725.54*** | ***7760.54*** |
|  | Logarithmic | 7993.56 | 8011.06 |
|  | Linear | 7980.13 | 7997.63 |
| **Accumbens** | ***Logistic*** | ***7178.88*** | ***7213.57*** |
|  | Logarithmic | 7191.16 | 7208.51 |
|  | Linear | 7205.67 | 7222.67 |

*Note:* Abbreviations: Akaike Information Criterion (AIC); Bayesian Information Criterion (BIC). The best-fitting models are shown in bold italics.

**Supplementary Table 5.**

NLMM parameter estimates for total brain volume development.

|  | **Estimate** | ***SE*** | **CV (%)** | ***p*** |
| --- | --- | --- | --- | --- |
| **Total brain volume** |  |  |  |  |
| Lower Asymptote | 1131.32 | 19.48 | 1.72 | - |
| $b$Sex(Lower Asymptote) | 200.60 | 26.16 | 13.04 | < .001 |
| Upper Asymptote | 1221.85 | 12.07 | 0.99 | - |
| $b$Sex(Upper Asymptote) | 103.46 | 20.46 | 19.78 | <.001 |
| Inflection | 16.20 | 0.65 | 4.01 | - |
| $b$Sex(Inflection) | -4.32 | 1.05 | 24.35 | <.001 |
| Hill | -2.86 | 2.88 | 100.74 | - |
| $b$Sex(Hill) | -1.87 | 2.97 | 159.10 | 0.53 |
| $b$Motion(Hill) | 0.06 | 0.02 | 37.21 | 0.007 |

*Note:* Parameter estimates are presented from the logistic model. Females are the reference group for sex covariate parameters. *SE* = Standard Error. CV = Coefficient of Variation

**Supplementary Table 6.**

Logistic model parameter estimates in analyses without covariates for the accumbens and amygdala.

| **Structure** | **Estimate** | ***SE*** | **CV (%)** |
| --- | --- | --- | --- |
| **Accumbens** |  |  |  |
| Lower Asymptote | 479.10 | 22.39 | 4.70 |
| Upper Asymptote | 711.90 | 34.42 | 4.80 |
| Inflection point | 10.20 | 1.23 | 12.00 |
| Hill | -2.20 | 0.49 | 22.70 |
| **Amygdala** |  |  |  |
| Lower Asymptote | 1580.70 | 46.34 | 2.90 |
| Upper Asymptote | 1865.50 | 25.63 | 1.40 |
| Inflection point | 7.80 | 0.87 | 11.20 |
| Hill | 2.80 | 0.53 | 19.00 |

*Note:* *SE* = Standard Error. CV = Coefficient of Variation.

**Supplementary Table 7.**

Model fit comparison in analyses without covariates.

| **Structure** | **Model** | **AIC** | **BIC** |
| --- | --- | --- | --- |
| **Cortical grey matter** | Linear | 9260.19 | 9272.69 |
|  | ***Logistic*** | ***9038.81*** | ***9061.31*** |
|  | Logarithmic | 9836.76 | 9849.26 |
| **White matter** | Linear | 8109.80 | 8122.30 |
|  | ***Logistic*** | ***7980.55*** | ***8003.05*** |
|  | Logarithmic | 8424.16 | 8436.66 |
| **Hippocampus** | ***Linear*** | ***8509.58*** | ***8522.08*** |
|  | Logistic | 8523.41 | 8545.91 |
|  | Logarithmic | 8543.59 | 8556.09 |
| **Amygdala** | Linear | 7710.69 | 7723.19 |
|  | ***Logistic*** | ***7684.71*** | ***7707.21*** |
|  | Logarithmic | 7715.91 | 7728.41 |
| **Caudate** | ***Linear*** | ***8287.03*** | ***8299.53*** |
|  | Logistic | 8433.94 | 8456.44 |
|  | Logarithmic | 8793.81 | 8806.30 |
| **Putamen** | ***Linear*** | ***8929.07*** | ***8941.57*** |
|  | Logistic | 8999.55 | 9022.04 |
|  | Logarithmic | 9255.04 | 9267.54 |
| **Pallidum** | Linear | 7986.77 | 7999.27 |
|  | ***Logistic*** | ***7777.29*** | ***7799.79*** |
|  | Logarithmic | 7993.22 | 8005.72 |
| **Accumbens** | Linear | 7217.43 | 7229.82 |
|  | ***Logistic*** | ***7188.91*** | ***7211.21*** |
|  | Logarithmic | 7213.60 | 7225.99 |
| **Thalamus** | ***Linear*** | ***9252.12*** | ***9264.62*** |
|  | Logistic | 9281.24 | 9303.74 |
|  | Logarithmic | 9675.39 | 9687.89 |

*Note:* The best-fitting model is shown in bold italics. For the amygdala, the logistic model was the best fitting model in analyses without covariates. AIC = Akaike Information Criterion. BIC = Bayesian Information Criterion.

**Supplementary Table 8.**

GAMM estimates for age and sex effects for global and subcortical brain regions, controlling for estimated total intracranial volume (eTIV) in analyses.

| **Structure** | **Effect** | **Statistics** | | | |
| --- | --- | --- | --- | --- | --- |
| **Cortical grey matter** | **Main effect** | **Estimate** | **SE** | ***t*** | ***p*** |
|  | Sex | 13103.42 | 4001.80 | 3.27 | 0.001 |
|  | **Trajectory** | **edf** | **Ref df** | ***F*** | ***p*** |
|  | S(age) | 5.55 | 5.55 | 659.88 | <.001 |
|  | S(age): sex | 4.54 | 4.54 | 20.77 | <.001 |
| **White matter** | **Main effect** | **Estimate** | **SE** | ***t*** | ***p*** |
|  | Sex | 6464.67 | 4168.34 | 1.55 | 0.12 |
|  | **Trajectory** | **edf** | **Ref df** | ***F*** | ***p*** |
|  | S(age) | 5.30 | 5.30 | 488.39 | <.001 |
|  | S(age): sex | 4.78 | 4.78 | 33.52 | <.001 |
|  |  |  |  |  |  |
| **Total brain volume** | **Main effect** | **Estimate** | **SE** | ***t*** | ***p*** |
|  | Sex | 22050.97 | 6205.30 | 3.55 | <.001 |
|  | **Trajectory** | **edf** | **Ref df** | ***F*** | ***p*** |
|  | S(age) | 5.54 | 5.54 | 214.03 | <.001 |
|  | S(age): sex | 4.88 | 4.88 | 38.74 | <.001 |
| **Hippocampus** | **Main effect** | **Estimate** | **SE** | ***t*** | ***p*** |
|  | Sex | -16.58 | 50.13 | -0.33 | 0.74 |
|  | **Trajectory** | **edf** | **Ref df** | ***F*** | ***p*** |
|  | S(age) | 4.48 | 4.48 | 23.23 | <.001 |
|  | S(age): sex | 4.04 | 4.04 | 18.82 | <.001 |
| **Amygdala** | **Main effect** | **Estimate** | **SE** | ***t*** | ***p*** |
|  | Sex | 58.96 | 23.62 | 2.50 | 0.01 |
|  | **Trajectory** | **edf** | **Ref df** | ***F*** | ***p*** |
|  | S(age) | 3.70 | 3.70 | 35.52 | <.001 |
|  | S(age): sex | 1.19 | 1.19 | 7.99 | 0.005 |
| **Caudate** | **Main effect** | **Estimate** | **SE** | ***t*** | ***p*** |
|  | Sex | -58.55 | 66.62 | -0.88 | 0.38 |
|  | **Trajectory** | **edf** | **Ref df** | ***F*** | ***p*** |
|  | S(age) | 5.19 | 5.19 | 270.19 | <.001 |
|  | S(age): sex | 4.07 | 4.07 | 27.64 | <.001 |
|  |  |  |  |  |  |
| **Putamen** | **Main effect** | **Estimate** | **SE** | ***t*** | ***p*** |
|  | Sex | 155.06 | 84.16 | 1.84 | 0.07 |
|  | **Trajectory** | **edf** | **Ref df** | ***F*** | ***p*** |
|  | S(age) | 4.75 | 4.75 | 246.91 | <.001 |
|  | S(age): sex | 1.00 | 1.00 | 57.60 | <.001 |
| **Pallidum** | **Main effect** | **Estimate** | **SE** | ***t*** | ***p*** |
|  | Sex | 47.33 | 33.38 | 1.42 | 0.16 |
|  | **Trajectory** | **edf** | **Ref df** | ***F*** | ***p*** |
|  | S(age) | 4.84 | 4.84 | 307.36 | <.001 |
|  | S(age): sex | 4.80 | 4.80 | 21.20 | <.001 |
| **Accumbens** | **Main effect** | **Estimate** | **SE** | ***t*** | ***p*** |
|  | Sex | 15.77 | 13.08 | 1.21 | 0.23 |
|  | **Trajectory** | **edf** | **Ref df** | ***F*** | ***p*** |
|  | S(age) | 4.75 | 4.75 | 56.31 | <.001 |
|  | S(age): sex | 1.79 | 1.79 | 11.32 | <.001 |
| **Thalamus** | **Main effect** | **Estimate** | **SE** | ***t*** | ***p*** |
|  | Sex | 207.53 | 85.77 | 2.42 | 0.02 |
|  | **Trajectory** | **edf** | **Ref df** | ***F*** | ***p*** |
|  | S(age) | 5.02 | 5.02 | 62.53 | <.001 |
|  | S(age): sex | 1.77 | 1.77 | 12.86 | <.001 |

*Note:* Sex represents the difference in the intercept for males compared to females. S(age) represents the smooth function of age for females. S(age): sex represents an age-by-sex interaction – the difference in the age trajectory of males compared to females. For each main effect, Estimate, standard error (SE), *t*-value, and *p*-value are reported. Effective degrees of freedom (edf), reference degrees of freedom (Ref df), *F*-statistic, and *p*-value are reported for each smooth function.

**Supplementary Table 9A.**

NLMM parameter estimates including scanner software version as a covariate.

| **Structure** | **Estimate** | ***SE*** | **CV (%)** | ***p*** |
| --- | --- | --- | --- | --- |
| **Cortex** |  |  |  |  |
| Inflection | 13.85 | 0.49 | 3.60 | - |
| $b$Software(Inflection) | **0.38** | 0.20 | 54.30 | 0.07 |
| **White matter** |  |  |  |  |
| Inflection | **14.86** | 1.52 | 10.30 | - |
| $b$Software(Inflection) | **-0.91** | 0.65 | 71.70 | 0.16 |
| **Pallidum** |  |  |  |  |
| Inflection | **11.22** | 0.52 | 4.60 | - |
| $b$Software(Inflection) | **-0.05** | 0.23 | 424.50 | 0.81 |
| **Caudate** |  |  |  |  |
| Slope | **-22.60** | 3.40 | 15.20 | - |
| $b$Software(Slope) | **-4.30** | 1.50 | 34.80 | 0.004 |

*Note.* SE = Standard Error. CV = Coefficient of Variation.

**Supplementary Table 9B.**

GAMM estimates for the main effect of scanner software version.

| **Metric/structure** | **Main effect: Software** | **SE** | ***t*** | ***p*** |
| --- | --- | --- | --- | --- |
| Cortical Grey Matter | 1883 | 2506 | 0.75 | 0.45 |
| White Matter | 1652 | 1206 | 1.37 | 0.17 |
| Total Brain Volume | 3359 | 3386 | 0.86 | 0.39 |
| Hippocampus | 54.69 | 18.01 | 3.04 | 0.002 |
| Amygdala | 19.51 | 10.10 | 1.93 | 0.05 |
| Putamen | -33.03 | 23.88 | -1.38 | 0.17 |
| Caudate | 13.36 | 15.84 | 0.84 | 0.40 |
| Pallidum | 39.87 | 12.21 | 3.27 | 0.001 |
| Accumbens | 4.86 | 6.85 | 0.71 | 0.48 |
| Thalamus | 4.73 | 28.92 | 0.16 | 0.87 |

*Note.* Estimate, Standard Error (SE), *t*-value, and *p*-value are reported. Effective degrees of freedom (edf), reference degrees of freedom (Ref df), *F*-statistic, and *p*-value are reported for each smooth function.


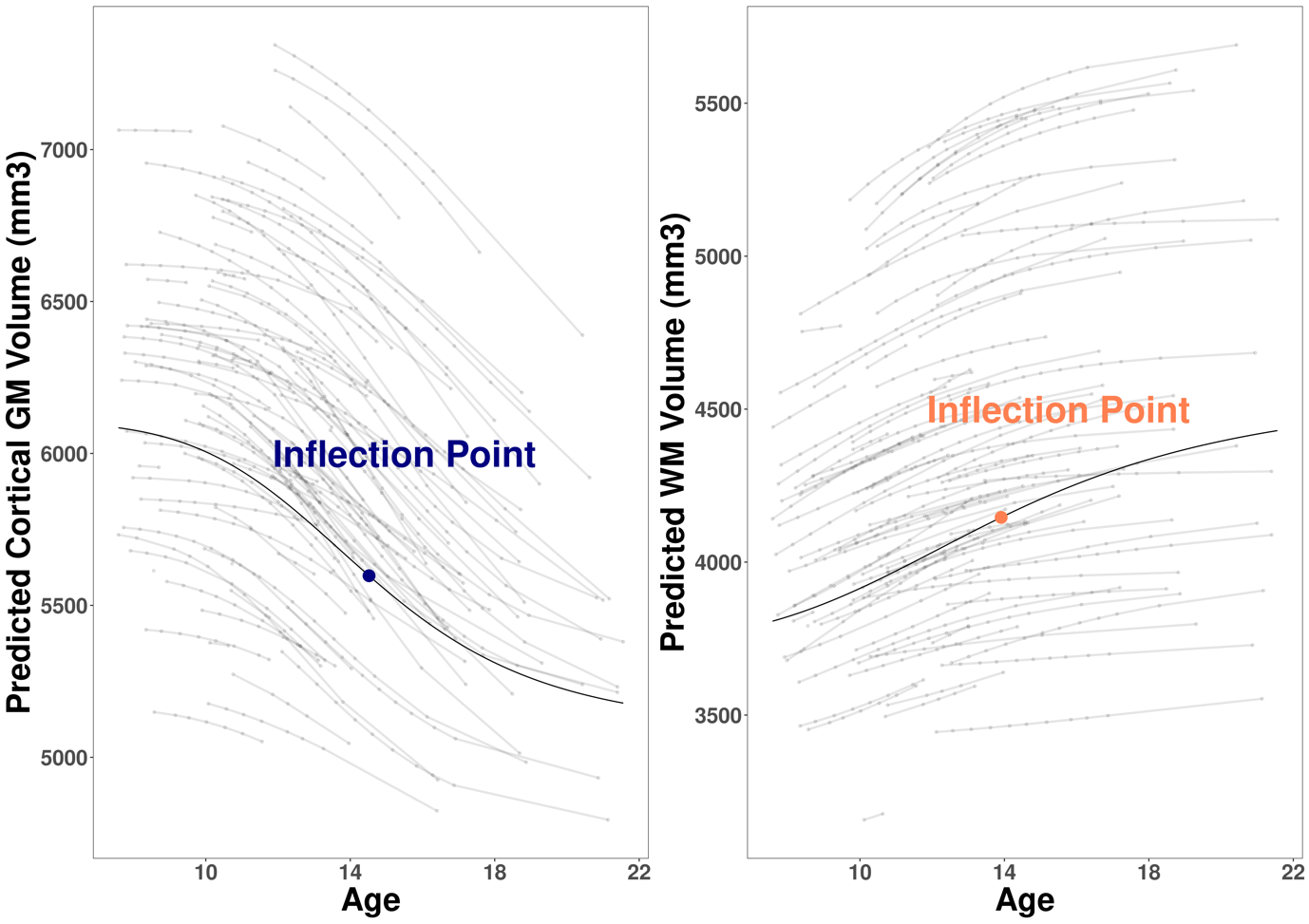


**Supplementary Figure 1.** Predicted cortical grey matter (GM) volume and white matter (WM) volume as a logistic function of age. The average trajectory with inflection points for cortical grey matter volume (14.52) and white matter volume (13.91) is overlaid on individual estimated trajectories.


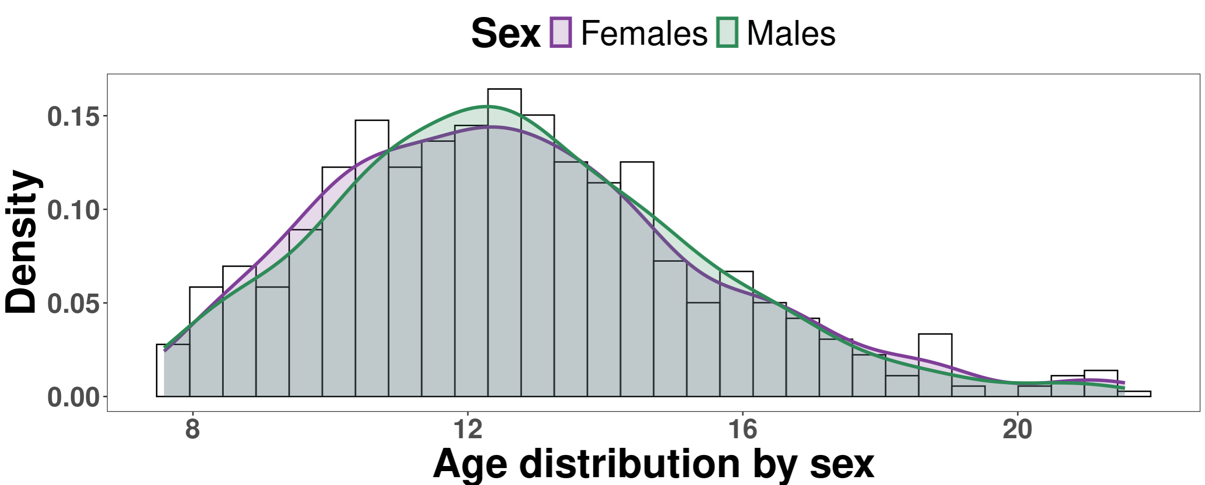


**Supplementary Figure 2.** Distribution of age in males and females.

1.
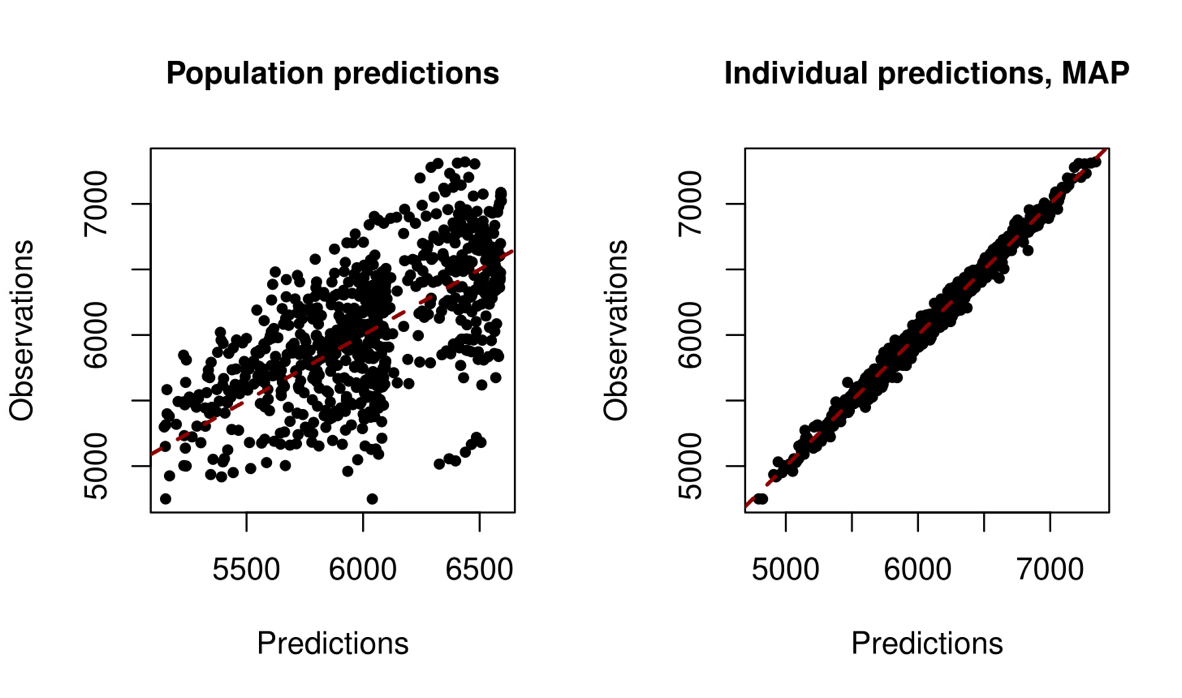
**Cortical grey matter volume**
2. **White matter volume**

**
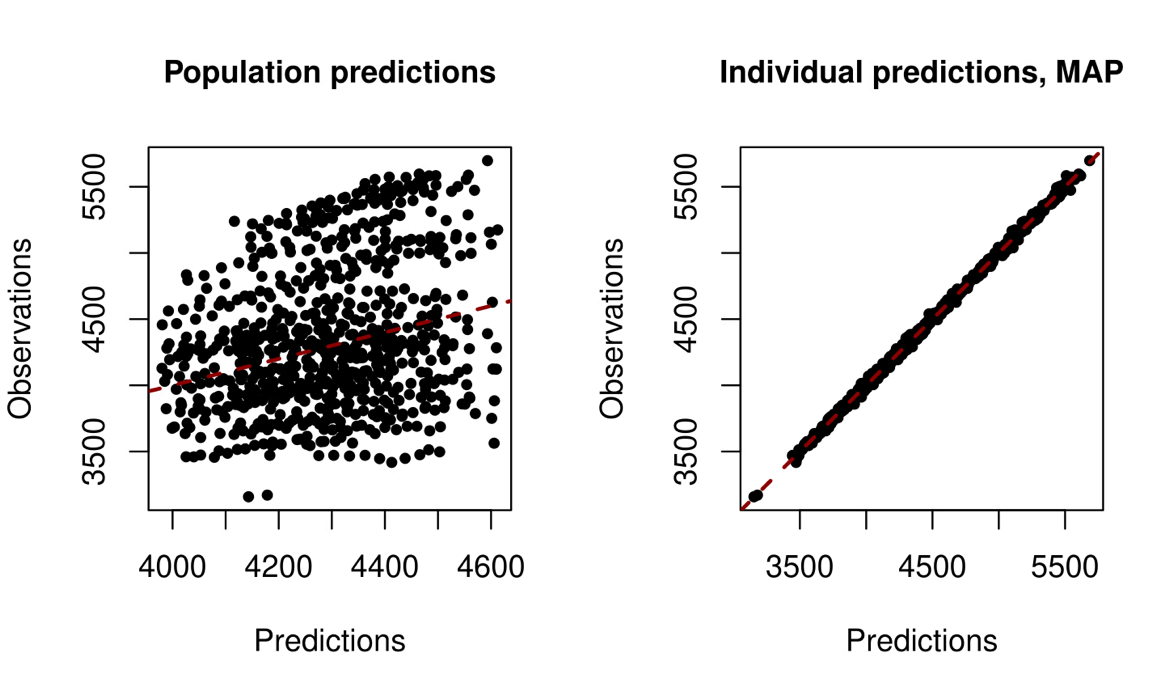
**

**Supplementary Figure 3.** Diagnostic plots for logistic model fit for cortical grey matter volume and white matter volume. Plots of observed vs. predicted values are displayed for population and individual predictions. MAP = Maximum A Posteriori estimation.

**
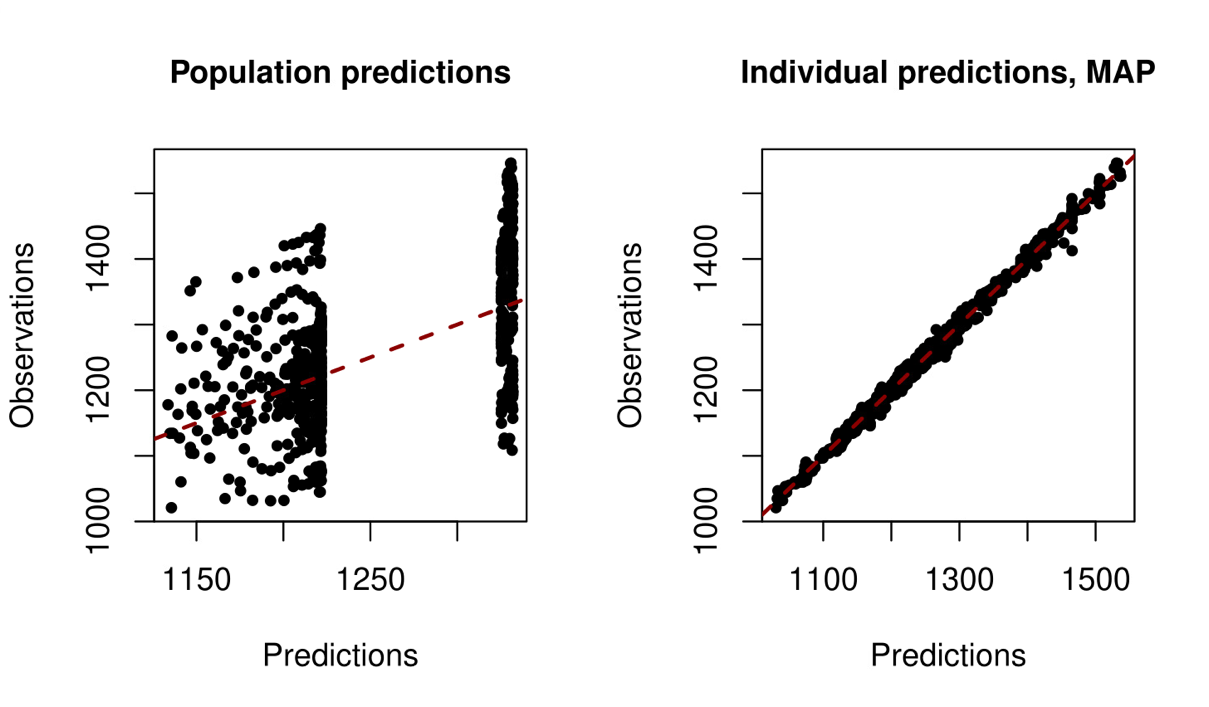
**

**Supplementary Figure 4.** Diagnostic plots for logistic model fit for total brain volume. The clustering of predicted values in the population predictions plot indicates poor model fit. MAP = Maximum A Posteriori estimation.

**
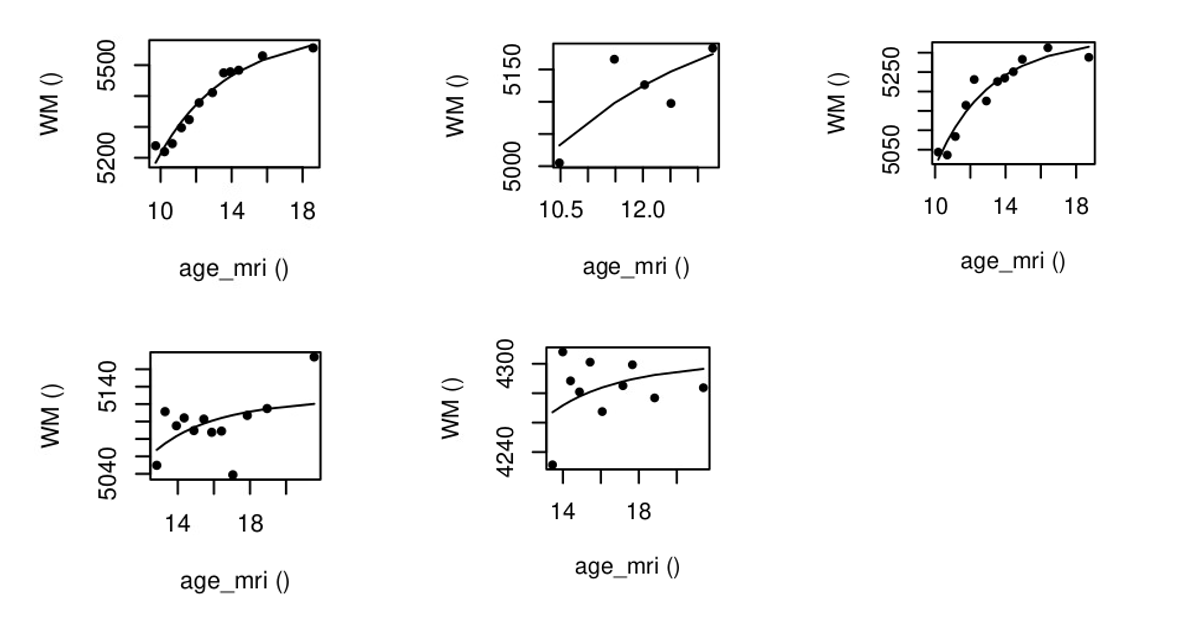
**

**Supplementary Figure 5.** Individual prediction plots for white matter volume development for the five participants with inflection point estimates earlier than the included age range. Observations (black dots) are overlaid on predicted trajectories (black line).


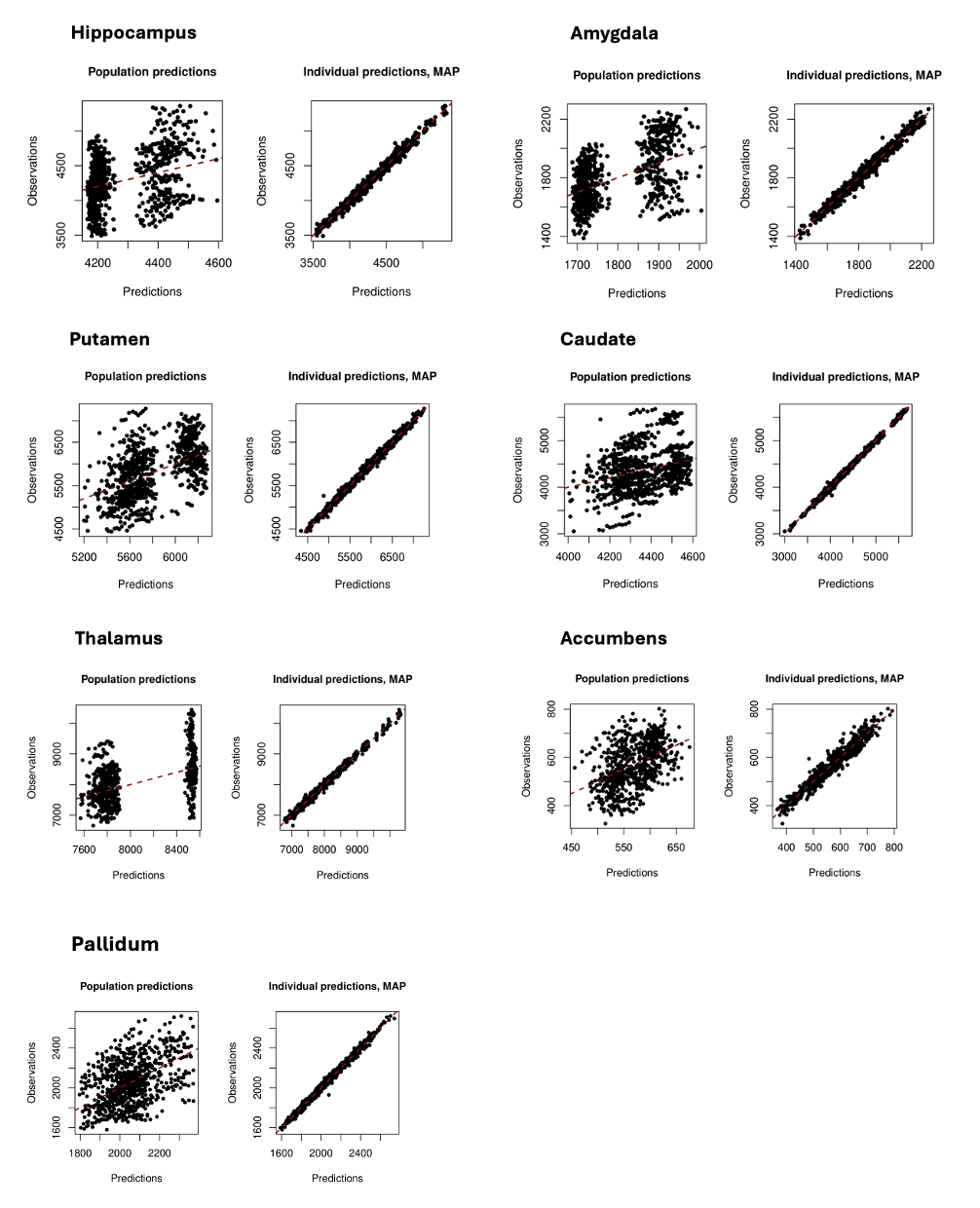
**Supplementary Figure 6.** Diagnostic plots for model fit for subcortical brain volumes. The hippocampus, amygdala, putamen, caudate, and thalamus were fit with a linear model. The accumbens and pallidum were fit with a logistic model. Plots of observed vs. predicted values are displayed for population and individual predictions. Distinct clustering in the hippocampus, amygdala, putamen and thalamus population prediction plots indicates potentially poor model fit. MAP = Maximum A Posteriori estimation.

**
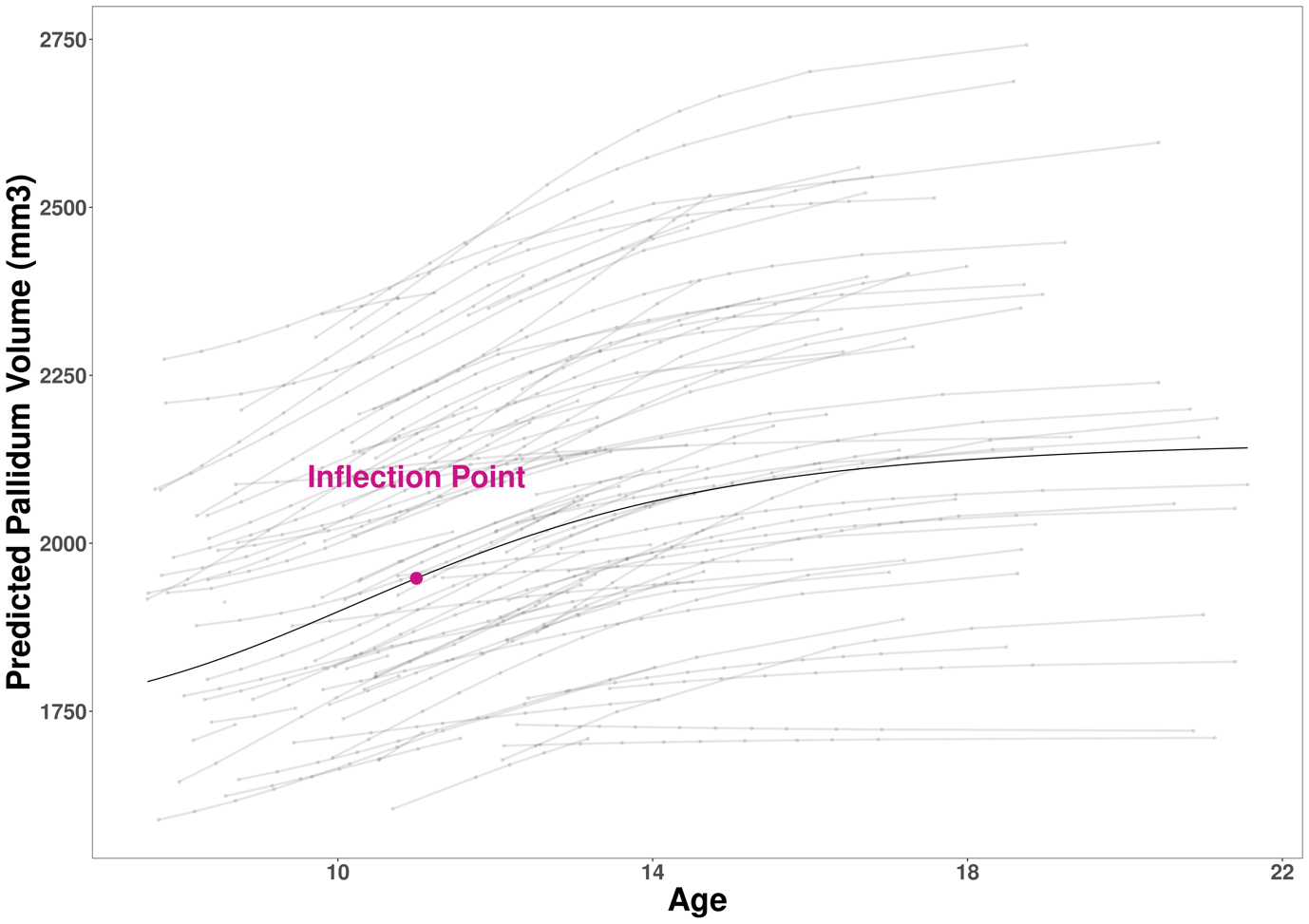
**

**Supplementary Figure 7**. Predicted pallidum volume as a logistic function of age. The average trajectory with inflection point (10.99 years) is overlaid on individual estimated trajectories. *Note*: The model estimate for the lower asymptote (1741.55 mm^3^) occurs earlier than the minimum age included in this sample (7.60 years) and is not visible in the above figure.

**
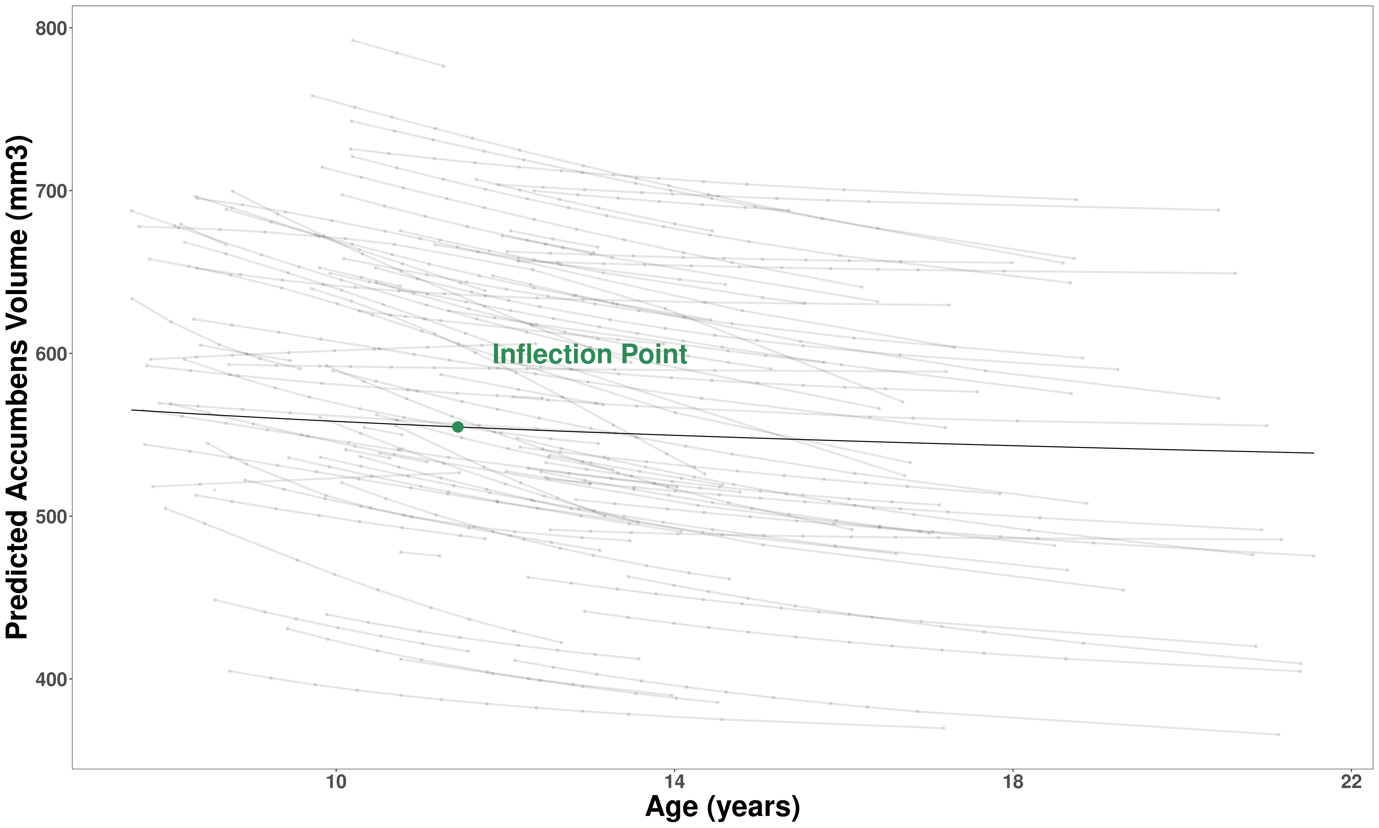
Supplementary Figure 8.** Predicted accumbens volume as a logistic function of age. *Note:* The model estimates for the upper and lower asymptotes (674.27 mm^3^ and 435.33 mm^3^) are not visible in the figure given that they correspond with ages outside of the included age-range in this study. Additionally, given the high CV value for the hill parameter (146.90), this developmental trend should be interpreted with caution.

**
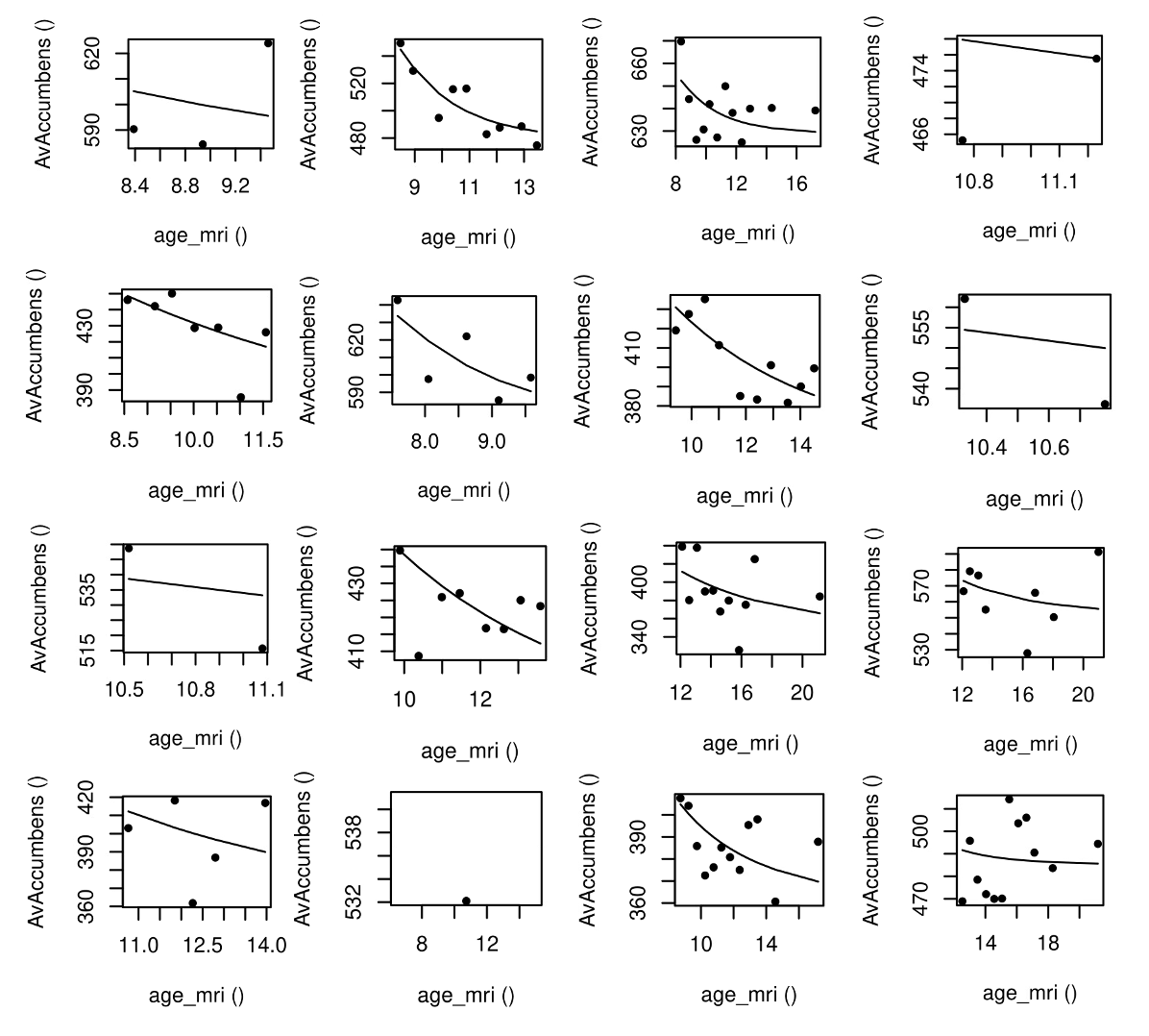
Supplementary Figure 9.** Individual prediction plots for 16 participants with inflection point values earlier than the included age-range for accumbens volume development. Individual observations (black dots) are overlaid on predicted trajectories (black line).
